# A novel strategy for classifying the output from an *in silico* vaccine discovery pipeline for eukaryotic pathogens using machine learning algorithms

**DOI:** 10.1186/1471-2105-14-315

**Published:** 2013-11-02

**Authors:** Stephen J Goodswen, Paul J Kennedy, John T Ellis

**Affiliations:** 1 School of Medical and Molecular Biosciences, ithree institute at the University of Technology Sydney (UTS), Sydney, Australia; 2 School of Software, Faculty of Engineering and Information Technology and the Centre for Quantum Computation and Intelligent Systems at the University of Technology Sydney (UTS), Sydney, Australia

## Abstract

**Background:**

An *in silico* vaccine discovery pipeline for eukaryotic pathogens typically consists of several computational tools to predict protein characteristics. The aim of the *in silico* approach to discovering subunit vaccines is to use predicted characteristics to identify proteins which are worthy of laboratory investigation. A major challenge is that these predictions are inherent with hidden inaccuracies and contradictions. This study focuses on how to reduce the number of false candidates using machine learning algorithms rather than relying on expensive laboratory validation. Proteins from *Toxoplasma gondii, Plasmodium sp*., and *Caenorhabditis elegans* were used as training and test datasets.

**Results:**

The results show that machine learning algorithms can effectively distinguish expected true from expected false vaccine candidates (with an average sensitivity and specificity of 0.97 and 0.98 respectively), for proteins observed to induce immune responses experimentally.

**Conclusions:**

Vaccine candidates from an *in silico* approach can only be truly validated in a laboratory. Given any *in silico* output and appropriate training data, the number of false candidates allocated for validation can be dramatically reduced using a pool of machine learning algorithms. This will ultimately save time and money in the laboratory.

## Background

This study addresses a major problem raised from a previous feasibility study [1] of a high-throughput *in silico* vaccine discovery pipeline for eukaryotic pathogens. A typical *in silico* pipeline output is a collection of different protein characteristics that are predicted by freely available bioinformatics programs [1]. These protein characteristics (referred henceforth as an evidence profile) represent potential evidence from which a researcher can make an informed decision as to a protein’s suitability as a vaccine candidate. The problem is that this evidence can be in different formats, contradicting, and inaccurate culminating in large numbers of false positive and negative decisions. The current solution is to accept that candidates will inevitably be missed due to the nature of an *in silico* approach and to rely on the laboratory validation to identify false candidates. The study herein focuses on how to reduce the false error rates using a computational approach.

Eukaryotic pathogens are extremely complicated systems comprised of thousands of unique proteins that are expressed in multifaceted life cycles and in response to varying environmental stimuli. A desired aim of an *in silico* approach for subunit vaccine discovery is to identify which of these proteins will evoke a protective, yet safe, immune response in the host [2,3]. It is currently impossible, however, to know within an *in silico* environment how a host will truly respond to a single protein or combination of proteins. Consequently, an *in silico* approach is not an attempt to replace experimental work but is a complementary approach to predict which proteins among thousands are worthy of further laboratory investigation. Vaccine discovery tools have been developed for prokaryotes [4,5], though, there is no *in silico* pipeline available to the public for eukaryotic pathogens and no clear consensus as to what type of protein constitutes an ideal subunit vaccine. Currently, the characteristics of proteins guaranteed to induce the desired immune response are poorly defined. Nevertheless, some protein characteristics which are considered relevant to vaccine discovery are sub-cellular location; presence of signal peptides, transmembrane domains, and epitopes [2,6-8].

The poor reliability of the *in silico* output arises because an unknown percentage of the *in silico* input (e.g. protein sequences, database annotations, and predicted evidence itself) are acknowledged incorrect or missing. Bioinformatics programs used to predict protein characteristics are, in general, inaccurate [9-15]. The inaccuracy can be a consequence of erroneous input data or overly simplistic algorithms, or simply due the complexity of the problem being solved. Since most prediction programs are imprecise, it can be expected that a percentage of the predicted protein characteristics will be incorrect. The difficulty encountered by a program user is to ascertain which of these predictions are correct and can contribute to the collection of evidence that supports a protein’s vaccine candidacy.

Given an *in silico* output, we propose that supervised machine learning methods can accurately classify the suitability of a protein, among potential thousands, for further laboratory investigation. Applying machine learning algorithms to solving biological problems is not novel. However, applying them to classify eukaryotic proteins for vaccine discovery is novel and this is reflected by the presence of only a few publications on the topic [16-18]. We illustrate the proposal on an *in silico* output comprising evidence from proteins experimentally shown to induce immune responses (referred henceforth as the benchmark dataset) and hence expected to be likely vaccine candidates.

## Results and discussion

Five datasets (see Table 1) containing evidence profiles were used in various ways to test the classification of a protein as either a vaccine candidate (YES classification) or non-vaccine candidate (NO classification). These evidence profiles for proteins from *Toxoplasma gondii, Neospora caninum, Plasmodium sp.*, and *Caenorhabditis elegans,* were compiled from the output predictions made by seven bioinformatics programs (see Table 2).

**Table 1:** Datasets used for training and testing machine learning models

**Name**^**a**^	**Number of proteins in each group**^**b**^			**Organism**	**Comments**
	**Membrane-associated**	**Secreted**	**Neither membrane-associated nor secreted**		
*T. gondii*	8	13	18	*Toxoplasma gondii*	
*Plasmodium*	47	26	51	*Plasmodium*	Includes *falciparum, yoelii yoelii, and berghei*
*C. elegans*	324	56	380	*Caenorhabditis elegans*	
Combined species	379	95	449	Combination of organisms	Includes *T. gondii, C. elegans, P. falciparum, P. yoelii yoelii*, and *P. berghei*
Benchmark	70^c^		70	Combination of two organisms	*T. gondii* and *Neospora caninum* (excludes the proteins in *T. gondii* dataset)

^a^This is the name used to refer to the dataset throughout the paper.

^b^Proteins (except for the benchmark dataset) were initially grouped in accordance with the subcellular location descriptor in UniProtKB, then fine-tuned in accordance to cross-validation testing, epitope presence, and reference to other UniProtKB annotations and Gene Ontology. Benchmark proteins were taken from published studies (70 experimentally shown to induce immune responses).

^c^Combination of proteins from membrane-associated, secreted, and unknown subcellular locations.

Note: Membrane-associated and Secreted proteins are expected ‘YES’ classification for vaccine candidacy. Neither membrane-associated nor secreted proteins are expected ‘NO’ classification. There was an attempt to create an equal representation of YES and NO classifications in the training datasets.

**Table 2:** High-throughput standalone programs used in this study to predict protein characteristics

**Name**	**Version**	**Predicted protein characteristic**	**URL (last viewed November 2013)**	**Published accuracy**^**a**^
WoLF PSORT	0.2	Protein localisation	http://wolfpsort.org/WoLFPSORT_package/version0.2/	80.0% [11]
SignalP	4.0	Secretory signal peptides	http://www.cbs.dtu.dk/services/SignalP/	93.0%^b^[9]
TargetP	1.1	Secretory signal peptides	http://www.cbs.dtu.dk/services/TargetP/	90.0% [10]
TMHMM	2.0	Transmembrane domains	http://www.cbs.dtu.dk/services/TMHMM/	97.0% [13]
Phobius	_	Transmembrane domains and signal peptides	http://phobius.binf.ku.dk/instructions.html	94.1% [12]
Peptide-MHC I Binding^c^		Peptide binding to MHC class I	http://tools.immuneepitope.org/main/html/download.html	95.7%^d^[14]
Peptide-MHC II Binding^c^		Peptide binding to MHC class II	http://tools.immuneepitope.org/main/html/download.html	76.0%^d^[15]

^a^Predictive accuracies taken from publications by the creators of the programs. The prediction accuracy varies for different target pathogens.

^b^SignalP version 3.0.

^c^Prediction Tools from The Immune Epitope Database and Analysis Resource (IEDB) [http://www.iedb.org].

^d^Area under curve value (AUC). Program uses different methods. For MHC I best method = artificial neural network (ANN) [14] and MHC II best method = Consensus [15].

**Figure 1:**
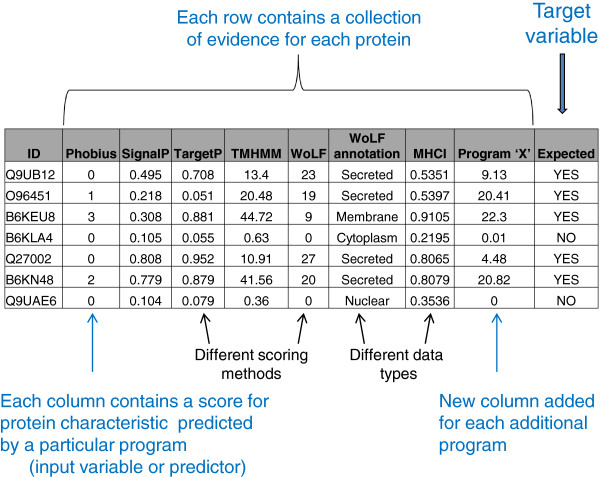
**A schematic of a typical *in silico *vaccine discovery pipeline output.** A typical *in silico* pipeline output is a collection of different protein characteristics that are predicted by bioinformatics programs. The schematic depicts a collection of some of the scores (potential evidence) associated with these predicted characteristics. A collection of scores for one protein is referred to as an evidence profile in the study. Each column represents a potential input variable or predictor for machine learning algorithms. The last column is a ‘YES’ or ‘NO’ as to whether the protein is expected to be a vaccine candidate (a requirement for machine learning training data) and represents the target variable i.e. the variable to be predicted for new profiles.

**Figure 2:**
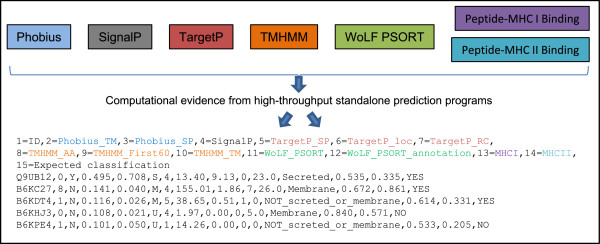
**An extract of evidence profiles.** Specific values from high-throughput standalone prediction programs are extracted and compiled to generate evidence profiles. Each row contains the collection of evidence for one protein (i.e. an evidence profile). Each column contains the score for a protein characteristic predicted by a specific program (i.e. an input variable or predictor). See the ‘Contents of evidence profiles’ subsection for a description of the columns. We apologise if the reintroduction of Figure 2 creates additional work for you, but hopefully you can appreciate the problem raised above, and ultimately the readers will benefit.

A typical profile is a mixture of data types corresponding to an accuracy measure, a perceived reliability, or a type of score for the protein characteristic being predicted (see Figure 1 and 2. There will always be considerable uncertainty in the profile due to inherent inaccuracies in the source of the evidence. That is, there is an unknown but expected percentage of inaccuracy in the input sequence, training data (if required), and program algorithm itself impeding precise prediction. This is irrespective of the target pathogen. The key question to be answered is whether we can classify potential vaccine candidates based on evidence profiles with hidden inaccuracies.

### Contents of evidence profiles

The Columns in the evidence profile are as follows: 1 = UniProt ID. 2 = Number of predicted transmembrane helices (Phobius_TM). 3 = A ‘Y’ or ‘N’ to indicate a predicted signal peptide (Phobius_SP) – a ‘Y’ is more likely to be a secreted protein. 4 = Probability of a secretory signal peptide (SignalP). 5 = Probability of a secretory signal peptide (TargetP_SP). 6 = Predicted localisation based on the scores: M = mitochondrion, S = secretory pathway, U = other location (TargetP_loc). 7 = Reliability class (RC) – from 1 (most reliable) to 5 (least reliable) and is a measure of prediction certainty (TargetP_RC). 8 = Expected number of amino acid residues in transmembrane helices (the higher the number the more likely the protein is membrane-associated) (TMHMM_AA). 9 = Expected number of residues in the transmembrane helices located in first 60 amino acids of protein. The larger the number the more likely the predicted transmembrane helix in the N-terminal is a signal peptide (TMHMM_First60). 10 = Number of predicted transmembrane helices (TMHMM_TM). 11 = Number of nearest neighbours that have a similar location (WoLF PSORT). 12 = Predicted subcellular location (Secreted or Membrane or NOT_secreted_or_membrane) (WoLF_ PSORT_annotation). 13 = Probability score encapsulating the collective potential of T-cell epitopes on protein with respect to vaccine candidacy (MHCI). Raw affinity scores derived from IEDB Peptide-MHC I Binding predictor. 14 = Probability score encapsulating the collective potential of T-cell epitopes on protein with respect to vaccine candidacy (MHCII). Raw affinity scores derived from IEDB Peptide-MHC II Binding predictor. 15 = Expected ‘YES’ or ‘NO’ vaccine candidacy (Target variable).

### Classifying with one individual piece of evidence

**Figure 3:**
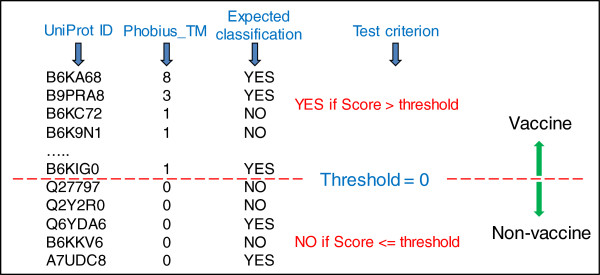
**Example of test applied to a predicted protein characteristic for the purpose of binary classification.** In this example, proteins are listed in descending order based on the number of transmembrane (TM) domains per protein predicted by the program Phobius (input value = Phobius_TM). A threshold value of 0 is applied to the score (i.e. number of TM domains) to segregate the list into two classifications. Above the threshold is ‘YES’ for vaccine candidacy and below or equal is ‘NO’. The classification is compared with the expected classification to determine sensitivity and specificity performance measures.

The first test was to determine whether proteins could be correctly classified using an individual piece of evidence (i.e. one input variable from an evidence profile). Figure 3 shows an example of how the test was applied. The sensitivity and specificity of the classification is shown in Table 3. The most notable observation is that non-vaccine candidates are predominantly correctly classified but the main trade-off is a substantial number of false negatives, as evidenced by the low sensitivity scores. The conclusion here is that there is no *one* individual input variable that can precisely determine the classification. This is not an unexpected result because each input variable represents only one particular protein characteristic and there is currently no *one* characteristic that conclusively epitomises a vaccine candidate.

**Table 3:** Sensitivity and specificity performance measures of binary classification for individual input variables taken from datasets

			**Datasets (comprising evidence profiles)**							
**Input variable**^**a**^	**Type**^**b**^	**Data**^**c**^	***T. gondii***		***Plasmodium***		***C. elegans***		**Benchmark**	
			**SN**	**SP**	**SN**	**SP**	**SN**	**SP**	**SN**	**SP**
Phobius_TM	TM	D	0.57	0.89	0.85	0.90	0.91	0.97	0.74	0.93
Phobius_SP	SP	T	0.52	0.89	0.39	1.00	0.25	0.99	0.49	0.96
SignalP	SP	C	0.52	1.00	0.39	1.00	0.25	1.00	0.39	1.00
TargetP_SP	SP	C	0.67	1.00	0.77	1.00	0.34	1.00	0.56	1.00
TargetP_loc	SP	T	0.67	0.94	0.76	1.00	0.27	1.00	0.56	1.00
TMHMM_AA	TM	C	0.62	0.89	0.66	0.98	0.91	1.00	0.80	1.00
TMHMM_First60	SP	C	0.43	0.93	0.26	1.00	0.37	1.00	0.49	0.97
TMHMM_TM	TM	D	0.57	0.89	0.65	1.00	0.90	1.00	0.77	1.00
WoLF_PSORT	Sub	C	0.76	0.94	0.42	1.00	0.77	0.98	0.60	0.97
WoLF_PSORT_annotation	Sub	T	1.00	0.56	0.92	0.74	1.00	0.72	0.96	0.73
MHCI	B	C	0.76	0.56	0.78	0.84	0.77	0.69	0.74	0.84
MHCII	B	C	0.86	0.39	0.80	0.74	0.90	0.52	0.54	0.84

*Abbreviations*: SN = sensitivity; SP = specificity; *T. gondii* = *Toxoplasma gondii*; *Plasmodium* = species in the genus *Plasmodium* including *falciparum, yoelii yoelii, and berghei*; *C. elegans* = *Caenorhabditis elegans*; Benchmark = dataset comprising evidence for *T. gondii* and *Neospora caninum* proteins from published studies.

^a^Input variable = predicted protein characteristic (i.e. a column from evidence profile).

^b^Type = prediction type: transmembrane domains (TM), secretory signal peptide (SP), sub-cellular location (Sub), peptide-MHC binding (B).

^c^Data = data type: discrete (D), continuous (C), text (T).

The values underlined denote the best performing input variable for classifying the published proteins.

Test criteria on input variable for binary classification:

Phobius_TM: YES if number of transmembrane domains > 0 else NO.

Phobius_SP: YES if = ‘Y’ else NO.

SignalP: YES if > 0.5 else NO.

TargetP_SP: YES if > 0.5 else NO.

TargetP_loc: YES if = ‘S’ else NO.

TMHMM_AA: YES if > 0 18^$$^ else NO.

TMHMM_ First60: YES if > 10^$$^ else NO.

TMHMM _TM: YES if number of transmembrane domains > 0 else NO.

Wolf_PSORT: YES if > 16^$$^ else NO.

WoLF_PSORT_annotation: YES if = ‘membrane’ or ‘secreted’ else NO.

MHCI: YES if > 0.5 else NO.

MHCII: YES if > 0.5 else NO.

^$$^A value recommended by the creator of the program.

$$\mathrm{Specificity}=\frac{\mathrm{True}\;\mathrm{Negatives}}{\mathrm{True}\;\mathrm{Negatives}+\mathrm{False}\;\mathrm{Positives}}$$

$$\mathrm{Sensitivity}=\frac{\mathrm{True}\;\mathrm{Positives}}{\mathrm{True}\;\mathrm{Positives}+\mathrm{False}\;\mathrm{Negatives}}$$

### Classifying with a rule-based approach

**Figure 4:**
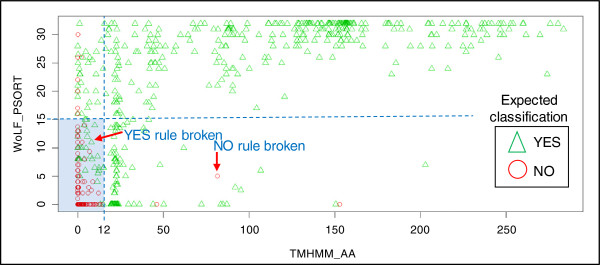
**A graph of proteins from the combined training dataset using only two input variables to illustrate a rule-based approach for binary classification.** Abbreviations: TMHMM_AA = number of amino acid residues in transmembrane helices (a transmembrane domain is expected to be greater than 18), WoLF PSORT = nearest neighbour score (16 = 50%). Triangles and circles indicate expected vaccine candidacy of proteins. The aim of the rule-based approach is to find the optimum threshold values that segregate majority of triangles from majority of circles. Best rule for binary classification is ‘NO if TMHMM_AA < 12 and WoLF PSORT < 15 (shaded area on graph) else YES’. Two examples of where YES and NO classification rules are broken are shown on graph. When this best rule was applied to the benchmark dataset the sensitivity and specificity were 0.43 and 0.97 respectively.

The next test was to determine if a combination of two or more input variables could efficiently perform the vaccine classification by applying an appropriate rule. Figure 4 illustrates the rule-based approach. A total of 17 combinations were tested with a programmed trial and error approach to obtain the maximum sensitivity and specificity. Table 4 shows the best rule from each combination. The best result achieved when tested on the benchmark dataset was 0.43 and 0.97 for sensitivity and specificity respectively. There were two main observations made from the rule-based testing: a rule that works well with one dataset does not necessarily generalise to another, and it is difficult to strike the ideal balance between sensitivity and specificity. For example, judicious adjustments to the rule threshold values can capture all proteins classified ‘YES’ in a test dataset (i.e. highly sensitive with zero false negatives) but at the expense of more false positives. Furthermore, if this adjusted rule is then applied to another dataset there are still false classifications. The conclusion here is that it is not feasible to compose a universal set of rules applicable to all datasets for the purpose of classifying proteins.

**Table 4:** Sensitivity and specificity of classifications on applying rule to benchmark dataset

**Rule description**	**SN**	**SP**
NO if TMHMM_AA < 12 and WoLF PSORT < 15 else YES	0.43	0.97
NO if TMHMM_TM = 0 and WoLF PSORT < 15 else YES	0.41	0.97
NO if Phobius_TM = 0 and WoLF PSORT < 15 else YES	0.41	0.90
NO if TMHMM_TM = 0 and MHCI < 0.5 else YES	0.63	0.84
NO if Phobius_TM = 0 and MHCII < 0.5 else YES	0.46	0.80
NO if TMHMM_AA < 18 and TargetP_SP < = 0.55 else YES	0.39	1.00
NO if TMHMM_TM = 0 and Target_SP < 0.55 else YES	0.31	1.00
NO if Phobius_TM = 0 and TargetP_SP < 0.45 else YES	0.34	0.93
NO if TMHMM_TM = 0 and SignalP < 3.8 else YES	0.24	1.00
NO if TMHMM_AA < 10 and SignalP < = 0.38 else YES	0.26	1.00
NO if TMHMM_AA < 12 and Phobius_SP = ‘N’ else YES	0.31	0.96
NO if TMHMM_TM = 0 and Phobius_SP = ‘N’ else YES	0.29	0.96
NO if TMHMM_AA < 18 and TargetP_SP < = 0.55 and MHCI < 0.5 else YES	0.31	0.84
NO if Phobius_TM = 0 and SignalP <0.45 else YES	0.21	0.93
NO if Phobius_TM = 0 and Phobius_SP = ‘N’ else YES	0.24	0.89
NO if TMHMM_AA < 18 and TargetP_SP < = 0.55 and WoLF_PSORT_annotation = NOT_screted_or_membrane else YES	0.37	0.73
NO if TMHMM_AA < 18 and TargetP_SP < = 0.55 and MHCII < 0.5 else YES	0.24	0.84

*Abbreviations*: SN = sensitivity; SP = specificity.

Note: In benchmark dataset, number of YES classifications = 70; number of NO classifications = 70; total number = 140.

### Classifying with machine learning algorithms

Seven, popular, supervised machine learning algorithms were used in an attempt to improve on the rule-based approach. Table 5 shows the sensitivity and specificity performance measures of the binary classification. The five datasets were used interchangeably for both training and testing. The table is presented as a matrix with training datasets in columns and test datasets in rows. For example, *T. gondii* dataset is used to build the decision tree model and tested on the benchmark dataset. Included in the matrix are classification results from cross-validation, which are expected to approach 1.0 (most algorithms have an inherent unavoidable error i.e. noise). Cross-validation results that greatly differ from 1.0 suggest there is at least one problematic evidence profile. The combined species dataset is the combination of the *T. gondii*, *Plasmodium,* and *C. elegans* datasets. The results, therefore, are positively biased when the combined species dataset is used for training and testing on datasets other than the benchmark. Similarly, testing on the combined species dataset with species-specific trained models is also positively biased. The main benchmark for the algorithm comparison is the classification of the benchmark proteins using the combined species to train the model.

**Table 5:** Sensitivity and specificity performance measures of binary classification on different test datasets when using machine learning algorithms with different training datasets

**Test dataset**	**Training dataset**									
	***T. gondii***		***Plasmodium***		***C. elegans***		**Combined species**		**Benchmark**	
	**SN**	**SP**	**SN**	**SP**	**SN**	**SP**	**SN**	**SP**	**SN**	**SP**
	**Decision Tree**^**a**^									
*T. gondii*	1.00^**b**^	0.81^**b**^	0.95	0.89	1.00	0.83	1.00	0.83	1.00	0.83
*Plasmodium*	0.84	0.90	1.00^**b**^	1.00^**b**^	0.85	0.96	1.00	0.92	1.00	0.98
*C. elegans*	0.87	0.93	1.00	0.99	1.00^**b**^	1.00^**b**^	1.00	0.99	1.00	0.98
Combined species	0.87	0.92	1.00	0.99	0.98	0.99	1.00^**b**^	0.98^**b**^	1.00	0.97
Benchmark	0.86	0.91	0.97	0.96	0.96	0.96	0.97	0.91	1.00^**b**^	1.00^**b**^
	**Adaptive boosting**^**a**^									
*T. gondii*	0.51^**b**^	0.06^**b**^	0.96	0.88	1.00	0.83	1.00	0.91	1.00	0.83
*Plasmodium*	0.82	0.99	0.98^**b**^	0.96^**b**^	0.95	0.96	1.00	1.00	1.00	0.98
*C. elegans*	0.87	0.99	1.00	1.00	1.00^**b**^	1.00^**b**^	1.00	1.00	1.00	0.98
Combined species	0.87	0.99	1.00	0.99	0.99	0.99	1.00^**b**^	0.99^**b**^	1.00	0.98
Benchmark	0.85	0.99	0.97	0.98	0.97	0.96	0.99	0.99	0.98^**b**^	0.97^**b**^
	**Random forest**^**a**^									
*T. gondii*	0.97^**b**^	0.90^**b**^	1.00	0.83	1.00	0.89	1.00	1.00	1.00	0.83
*Plasmodium*	0.87	1.00	0.99^**b**^	0.99^**b**^	1.00	1.00	1.00	1.00	1.00	0.98
*C. elegans*	0.83	1.00	0.98	1.00	1.00^**b**^	1.00^**b**^	1.00	1.00	1.00	1.00
Combined species	0.84	1.00	0.98	0.99	1.00	1.00	1.00^**b**^	1.00^**b**^	1.00	0.99
Benchmark	0.82	1.00	0.99	0.99	0.99	1.00	0.97	0.99	0.99^**b**^	0.99^**b**^
	**k-Nearest neighbour**									
*T. gondii*	0.80^**b**^	0.83^**b**^	1.00	0.83	0.95	0.83	1.00	0.83	0.90	0.78
*Plasmodium*	0.77	0.96	0.95^**b**^	0.84^**b**^	0.88	0.96	0.99	0.94	0.81	0.96
*C. elegans*	0.88	0.99	0.99	0.95	0.96^**b**^	0.98^**b**^	0.99	0.99	0.95	0.98
Combined species	0.87	0.98	0.99	0.94	0.97	0.98	0.96^**b**^	0.97^**b**^	0.92	0.97
Benchmark	0.93	0.96	1.00	0.90	0.96	0.96	0.96	0.97	0.98^**b**^	0.96^**b**^
	**Naive bayes classifier**									
*T.gondii*	1.00^**b**^	0.91^**b**^	1.00	0.78	1.00	0.83	1.00	0.83	1.00	0.83
*Plasmodium*	0.97	0.98	0.98^**b**^	0.99^**b**^	1.00	0.92	1.00	0.96	1.00	0.98
*C. elegans*	0.87	1.00	0.92	0.95	1.00^**b**^	0.98^**b**^	0.97	0.98	1.00	0.99
Combined species	0.89	0.99	0.93	0.95	1.00	0.97	0.98^**b**^	0.97^**b**^	1.00	0.98
Benchmark	0.81	1.00	0.97	0.94	1.00	0.93	1.00	0.99	1.00^**b**^	1.00^**b**^
	**Neural networks**^**a**^									
*T. gondii*	0.98^**b**^	0.90^**b**^	0.99	0.83	1.00	0.84	1.00	0.91	0.99	0.83
*Plasmodium*	0.88	0.92	0.99^**b**^	0.89^**b**^	0.99	0.97	0.97	0.98	0.93	0.97
*C. elegans*	0.83	0.99	0.92	0.98	0.99^**b**^	0.99^**b**^	1.00	1.00	0.98	0.97
Combined species	0.91	0.96	0.93	0.98	0.99	0.98	0.99^**b**^	0.98^**b**^	0.97	0.97
Benchmark	0.78	0.97	0.97	0.97	0.99	0.95	0.99	0.96	1.00^**b**^	0.95 ^**b**^
	**Support vector machines**									
*T.gondii*	0.83^**b**^	0.92^**b**^	0.89	1.00	0.89	0.89	1.00	0.89	1.00	0.83
*Plasmodium*	0.88	0.97	0.98^**b**^	0.98^**b**^	0.96	0.98	1.00	0.98	1.00	0.98
*C. elegans*	0.83	0.89	0.98	0.99	0.94^**b**^	0.99^**b**^	0.99	1.00	0.91	0.99
Combined species	0.84	0.91	0.98	0.98	0.99	0.99	0.92^**b**^	0.99^**b**^	0.93	0.98
Benchmark	0.74	0.99	0.96	0.96	0.94	0.99	0.96	1.00	0.83^**b**^	0.92^**b**^

*Abbreviations*: SN = sensitivity; SP = specificity; *T. gondii* = *Toxoplasma gondii*; *Plasmodium* = species in the genus *Plasmodium* including *falciparum, yoelii yoelii, and berghei*; *C. elegans* = *Caenorhabditis elegans*; Combined species = combination of *T. gondii*, *Plasmodium*, and *C. elegans* datasets; Benchmark = dataset comprising evidence for *T. gondii* and *Neospora caninum* proteins from published studies.

^**a**^Results from the same input data fluctuate. The algorithm-specific R functions were executed 100 times and the prediction outcomes (false positives and negatives, true positives and negatives) were averaged to calculate SN and SP.

^**b**^Obtained from multiple cross-validations i.e. the algorithm-specific R functions randomly used 70% of the training dataset to build a model and the remaining 30% was used in the binary classification test. The cross-validation was executed 100 times and the prediction outcomes were averaged to calculate SN and SP.

The values underlined denote the best performing training dataset for classifying the benchmark proteins.

In summary, the best benchmark performing algorithm (based on the sum of sensitivity and specificity) is naïve Bayes; then adaptive boosting; followed jointly by random forest and support vector machines (SVM); then neural networks, *k-*nearest neighbour, and finally decision tree. With the exception of decision tree, the difference in performance is so minimal that the ranked performance here could easily change given different training and test datasets and/or fine-tuning of the algorithm parameters. Ultimately, there was no apparent difference between the algorithms with respect to solving this specific problem of classifying evidence profiles.

### Factors affecting performance of machine learning algorithms

It is the content of the training dataset and in particular the number of problematic profiles in both the training and test datasets that have the greatest impact on the performance of the algorithm. Certain profiles are more problematic than others for some algorithms to classify and tend to be consistently misclassified. The *T. gondii* trained model performed the poorest when tested on the benchmark proteins irrespective of the algorithm used. It is tempting to assume that the poor performance from the *T. gondii* trained model was due to a misclassification of the target input variable for some of the evidence profiles. However, there are two other proposed reasons for this inaccuracy: the training dataset contains the least number of evidence profiles (39 in total), but more importantly it contains three labelled profiles with questionable evidence (i.e. erroneous evidence predictions identified when manually assessing them). Cross-validation is a useful indication that a particular profile is problematic. Problematic profiles, both in the training and test datasets, tend to contain ambiguous evidence which can cause the algorithm to make an unexpected classification. Based on cross-validation, the *T. gondii* data contained the most problematic profiles for all algorithms, followed by *Plasmodium*, benchmark and *C. elegans* datasets. Removing problematic profiles improves performance in cross-validation. It is therefore tempting to remove these problematic profiles from the training datasets for deployment but their removal negatively impacts performance. The motivation behind using the machine learning algorithms is to overcome the effects of erroneous evidence that is currently inherent in the *in silico* vaccine discovery output. Consequently, the training data *should* retain problematic profiles for building models for deployment. They need to be retained in the application of the model because it is unclear whether these problematic profiles are incorrect or whether they are correct but rare (i.e. they are outliers). New profiles for classification are expected to contain an unknown percentage of similar erroneous evidence. Algorithms vary in their ability to handle problematic profiles according to what other profiles are represented in the training dataset. For example, the combined species trained model is a collection of exactly the same profiles as those in the individual species trained models. However, the algorithms when trained with the combined species are able to correctly classify the problematic profiles more effectively than individual species trained models.

The results in Table 5 show that there is no fundamental difference between evidence profiles from different eukaryotic species. For example, the benchmark dataset is composed of *T.gondii* and *N. caninum* data and yet both the *Plasmodium* and *C. elegans* trained models outperformed the *T. gondii* trained model. The ideal training dataset for the classification problem described herein is one that contains the most variety of evidence profiles irrespective of the source species.

None of the algorithms can consistently classify evidence profiles without false predictions irrespective of the training dataset. Each algorithm nonetheless performed better than the rule-based approach with a collective average sensitivity and specificity of 0.97 and 0.98. The main reason why the machine learning algorithms performed better than the rule-based approach in this study is related to how they handle erroneous evidence. For example, a classification rule, applied to a combination of input variables, fails when only one input variable is erroneous. Machine learning algorithms, despite erroneous evidence in both the training and test datasets, can still exploit a generalised pattern within the collection of evidence for the purpose of classification.

### A proposed classification system

**Figure 5:**
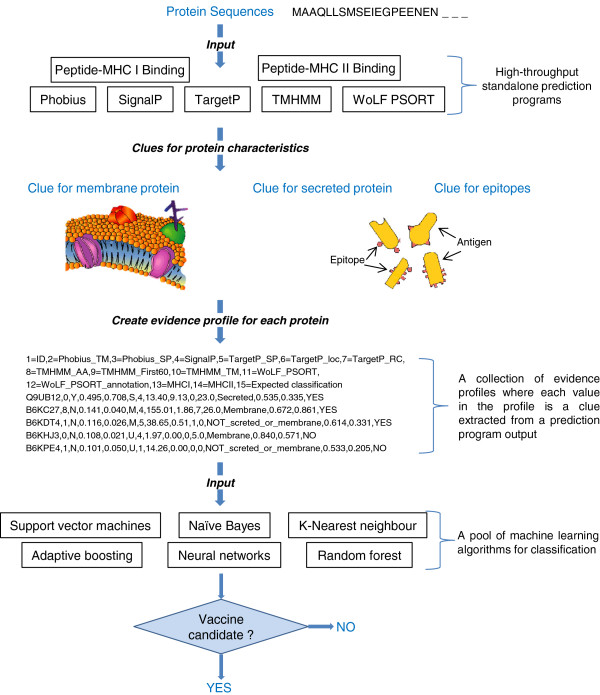
**Overview of a proposed classification system using a pool of machine learning algorithms to determine the suitability of proteins for vaccine candidacy.** Protein sequences for a target species are input into seven prediction programs. These programs provide evidence as to whether the proteins associated with the sequences are either membrane-associated or secreted, and contain epitopes. Evidence for each protein is collated to create an evidence profile. A collection of evidence profiles are used as input to a pool of six independent machine learning algorithms for classification. Final classification is based on voting and a majority rule decision.

The proposed classification system (see Figure 5) uses the ensemble of classifiers, excluding the decision tree, to make a final classification based on voting and a majority rule decision from predictions of the individual classifiers. In the case of a tied vote, the decision is deemed a YES classification. The logic behind this decision is that false positives are preferential to false negatives as they can be identified later during the laboratory validation. Table 6 shows the UniProt identifier for proteins from the benchmark dataset that were consistently incorrectly classified by the machine learning algorithms. At least one of the six algorithms failed to correctly classify six proteins (Q27298, B0LUH4, P84343, Q9U483, B9PRX5, B9QH60) that were expected to be YES and three proteins (B6K9N1, B9Q0C2, B9PK71) expected to be NO. Table 7 provides a description of these misclassified proteins. After applying the majority rule approach, all proteins were classified as expected. The final predicted classification of protein Q27298 was YES based on a tied decision. There are three possible reasons why a protein in the final classification process might be misclassified: 1) the expected classification is incorrect, 2) the majority of algorithms fail, and 3) the evidence profile is too problematic. The misclassifications in Table 6 suggest that they were mainly due to the failure of a particular algorithm when considering the successful classification by other algorithms. The evidence profiles for Q27298 and B9PRX5 are possibly problematic for the algorithms that made the misclassification. This is most likely because the algorithms have not been trained for a profile of this type i.e. the training dataset is failing. In this case (or in the case of any classified vaccine candidate), false positives can only be identified in the laboratory. Interpreting the relationship between evidence profiles and an immune response in host remains a challenge to the *in silico* vaccine discovery approach.

**Table 6:** Misclassified proteins from the benchmark dataset by machine learning algorithms

**Algorithm**	**Incorrect YES classifications**	**Incorrect NO classifications**
Adaptive boosting		Q27298
*k*-Nearest Neighbour	B6K9N1	B0LUH4
	B9Q0C2	P84343
		Q9U483
Naive Bayes Classifier	B9PK71	
Neural Networks		
Random Forest		Q27298
		B9PRX5
Support Vector Machines		Q27298
		B9QH60
		B9PRX5

Protein identifiers e.g. Q27298 are UniProt IDs. Refer to Additional file 1 for a description of the protein and its relevance as a vaccine candidate.

**Table 7:** Description of proteins from the benchmark dataset that were misclassified by at least one machine learning algorithm

**UniProt ID**	**Protein name**	**Subcellular annotation**	**Expected classification**	**Final classification**^**a**^	**Misclassification by algorithm**^**b**^	**Evidence profile**^**c**^
Q27298	SAG1 protein (P30	Membrane	YES	YES	AB RF SVM	Q27298,0,Y,0.297,0.141,M,2,7.30,0.56,0,21.5,Secreted,0.255,0.205,YES
B0LUH4	Microneme protein 13	Unknown	YES	YES	kNN	B0LUH4,0,Y,0.888,0.907,S,1,0.11,0.11,0,29.0,Secreted,0.270,0.355,YES
P84343	Peptidyl-prolyl cis-trans isomerase	Unknown	YES	YES	kNN	P84343,0,Y,0.817,0.963,S,1,1.11,1.11,0,29.0,Secreted,0.465,0.536,YES
Q9U483	Microneme protein Nc-P38	Unknown	YES	YES	kNN	Q9U483,0,Y,0.427,0.587,S,4,0.23,0.23,0,30.0,Secreted,0.355,0.1736,YES
B9PRX5	Proteasome subunit alpha type	Unknown	YES	YES	RF SVM	B9PRX5,0,Y,0.250,0.254,M,2,16.81,7.23,0,22.0,Secreted,0.648,0.515,YES
B9QH60	Acetyl-CoA carboxylase, putative	Unknown	YES	YES	SVM	B9QH60,1,N,0.322,0.019,M,1,22.02,0.00,1,5.0,Secreted,0.846,0.437,YES
B6K9N1	Cytochrome P450 (putative)	Unknown	NO	NO	kNN	B6K9N1,1,N,0.131,0.041,U,2,15.35,0.03,0,5.0,Membrane,0.197,0.480,NO
B9Q0C2	Anamorsin homolog	Cytoplasm	NO	NO	kNN	B9Q0C2,0,Y,0.245,0.108,U,4,0.54,0.00,0,20.0,Secreted,0.382,0.210,NO
B9PK71	DNA-directed RNA polymerase subunit	Nucleus	NO	NO	NB	B9PK71,0,N,0.188,0.223,U,4,0.00,0.00,0,22.0,Secreted,0.368,0.380,NO

^a^Final classification takes into account predictions from each algorithm and the most frequent classification type is used i.e. a majority rule approach. A YES classification is adopted for tied votes e.g. Q27298.

^b^Algorithms are executed multiple times on the same input data. An in-house Perl script summarises the multiple runs and indicates the number of times (as a percentage) the predicted classification of protein differs from the expected. Proteins are regarded as misclassified if the number of times = 100%.

^c^Column headers: 1 = ID, 2 = Phobius_TM, 3 = Phobius_SP, 4 = SignalP, 5 = TargetP_SP, 6 = TargetP_loc, 7 = TargetP_RC, 8 = TMHMM_AA, 9 = TMHMM_First60, 10 = TMHMM_TM, 11 = WoLF_PSORT, 12 = WoLF_PSORT_annotation, 13 = MHCI, 14 = MHCII, 15 = Expected classification.

*Abbreviations*: AB = Adaptive boosting, RF = random forest, SVM = support vector machines, NB = Naive Bayes, kNN = *k*-Nearest neighbour, NN = neural network.

### Future developments

The outcome of the classification system is a list of proteins that are worthy of laboratory investigation. Each protein in the list is assumed to have an equal chance of being a vaccine candidate. An improvement to the proposed classification system is to score the proteins according to a likelihood or confidence level that the classifications are correct. The R functions for SVM and random forest support class-probabilities i.e. an estimated probability for each protein belonging to ‘YES’ and ‘NO’ classes. For such an extension, the format of the training datasets are the same except the target value would no longer be a ‘YES’ or ‘NO’ but a single probability score that attempts to encapsulate each snippet of evidence representing the evidence profile. Determining such a score is a challenge that still remains. The advantage of an appropriate scoring system is that the proteins in the vaccine candidacy list can then be ranked. A caveat here is that the ranking is based on a confidence level of prediction. A protein with a high probability score does not necessarily imply a high probability of an immune response when injected in a host.

The proposed classification system is intended to illustrate a framework on which researchers can build more efficient systems. For example, only seven high-throughput prediction programs were used here to create the evidence profiles. There are other bioinformatics programs [1] that could be used to predict similar or additional protein characteristics from protein sequences, such as GPI anchoring, molecular function, and biological process involvement. At the time of writing, there is no high-throughput standalone GPI predictor. Appropriate values that support vaccine candidacy could be extracted from these extra program outputs and added to the evidence profile as additional columns in the training datasets.

There are examples of proteins with annotated interior subcellular locations that have been observed to induce an immune response [19]. It is assumed here that these proteins are not naturally exposed to the immune system but were exposed as a consequence of experimental conditions. Nevertheless, the important point here is that they *do* induce an immune response and are potential vaccine candidates. These interior proteins are missed by the current proposed classification system. All protein types that induce an immune response in theory need to be addressed to create a totally encompassing system for *in silico* vaccine discovery. This can only be accomplished if distinguishing characteristics that exemplify antigenicity can be predicted given proteins sequences. A prediction program that distinguishes antigenic and non-antigenic interior proteins is sought.

## Conclusion

We conclude the following when given a high-throughput *in silico* vaccine discovery output consisting of predicted protein characteristics (evidence profiles) from thousands of proteins: 1) machine learning algorithms can perform binary classification (i.e. yes or no vaccine candidacy) for these proteins more accurately than human generated rules; 2) there is no apparent difference in performance (i.e. sensitivity and specificity) between the algorithms; adaptive boosting, random forest, *k*-nearest neighbour classifier, naive Bayes classifier, neural networks, and SVM, when performing this particular classification task; 3) none of the algorithms can consistently classify evidence profiles without false predictions using the training datasets in this study; 4) there is no fundamental difference in patterns in evidence profiles compiled from different species e.g. a model trained on one species can classify proteins from another and hence no target specific training datasets are required; 5) an ideal training dataset is one that contains the most variety of evidence profiles irrespective of the source species e.g. quality and variety are indisputably the most important factors that impact the accuracy of algorithms; and 6) a pool of algorithms with a voting and majority rule decision can perform classification with a high degree of accuracy e.g. 100% sensitivity and specificity was demonstrated in this study by correctly determining the *expected* classification of the benchmark dataset.

Vaccine candidates from an *in silico* approach can only be truly validated in a laboratory. There are essentially two options. One is to rely on laboratory validation to identify false candidates. The other is to use our proposed classification system to identify those proteins more worthy of laboratory validation. This will ultimately save time and money by reducing the false candidates allocated for validation.

## Methods

### Eukaryotic pathogens used in study

*Toxoplasma gondii, Plasmodium sp.*, and *Caenorhabditis elegans* were the chosen species to train the machine learning algorithms. *Toxoplasma gondii* is an apicomplexan pathogen responsible for birth defects in humans [20] and is an important model system for the phylum Apicomplexa [21-23]. Species in the genus *Plasmodium* are also apicomplexan pathogens and can cause the disease malaria [24]. These species were selected because in comparison to most other pathogens, they have experimentally validated data for protein subcellular location, albeit limited for *T. gondii*. *Caenorhabditis elegans* is a free-living nematode that is not a pathogen but is rich in validated data [25]. This species was particularly chosen to investigate whether a universal training dataset could be used for the classification of proteins from any eukaryotic pathogen or whether target specific training datasets are required.

### Training data for machine learning algorithms

Two sets of distinct evidence profiles for each training dataset were required. One set representing evidence for proteins that are vaccine candidates and another for non-vaccine candidates. The major challenge here is that there are too few examples of protein subunit vaccines, irrespective of the target pathogen, to create ideal training datasets. Consequently, the training datasets used in this study are based on proteins that are only likely vaccine candidates – ‘likely’ in this context is based on two *a priori* held hypotheses*:*1) a protein that is either external to or located on, or in, the membrane of a pathogen is more likely to be accessible to surveillance by the immune system than a protein within the interior of a pathogen [26]; and 2) a protein containing peptides (T-cell epitopes) that bind to major histocompatibility complex (MHC) molecules fulfils one of several prerequisites for a vaccine based on this protein. That is, a protein vaccine candidate needs to contain T-cell epitopes to induce the creation of a memory T-cell repertoire capable of recognizing a pathogen [27,28].

Appropriate protein sequences for *T. gondii*, *C. elegans*, and *Plasmodium* species were downloaded from the Universal Protein Resource knowledgebase (UniProtKB at http://www.uniprot.org/). In UniProtKB at the time of writing, there were 19261 proteins for *T. gondii* species (this includes strains such as ME49, VEG, RH, and GT1), 25765 for *C. elgans,* and 75,507 for the genus *Plasmodium*. Despite *T. gondii* being a well-studied organism, only 55 proteins had the status of manually annotated and reviewed. In comparison, *C. elegans* had 3360 reviewed and *Plasmodium* 488. A challenge was that the protein’s annotations in UniProtKB (e.g. protein name, domains, protein families, subcellular location etcetera) were not necessarily indicative to selecting the desired three classes of proteins: secreted, membrane-associated, and other. The subcellular location annotation was the most informative out of all annotations. Of the reviewed proteins, 39 for *T. gondii*, 1190 for *C. elegans* and 202 for *Plasmodium* had experimental evidence to support the annotation for their subcellular location. To aid in creating a preliminary training dataset, proteins from the desired subcellular locations were selected using the advanced search facility in UniProt and entering either a partial or whole term in the subcellular location field. Using the word ‘membrane’ in the UniProt advanced search, 11 of the 39 *T. gondii* proteins were selected. Similarly, 10 out of 39 were selected using the word ‘secreted’. For *C. elegans,* 796 of the 1190 proteins with experimentally derived subcellular locations had the word ‘membrane’ and 47 had ‘secreted’ (unlike apicomplexan pathogens, *C. elegans* do not secrete proteins for the purpose of invasion and survival within host cells). There were only four *Plasmodium* proteins with ‘secreted’ annotation in contrast to 134 with membrane (there are many more secreted proteins in UniProtKB but not yet reviewed). This broad word search selected undesired proteins with subcellular descriptions such as parasitophorous vacuole membrane and golgi apparatus membrane. Proteins with inappropriate subcellular descriptions were manually removed or reclassified in the training datasets on consultation with the UniProt controlled vocabulary (http://www.uniprot.org/docs/subcell). The expected ‘YES’ or ‘NO’ classification for each protein in the training datasets was fined-tuned in accordance to cross-validation testing, epitope presence as per reference to the Immune Epitope Database and Analysis Resource (http://www.iedb.org), and reference to other UniProtKB annotations and Gene Ontology. Descriptions of the datasets are shown in Table 1.

### Bioinformatics prediction programs

The downloaded protein sequences from UniProtKB were used as input to seven prediction programs (WoLF PSORT [11], SignalP [29], TargetP [10], TMHMM [13], Phobius [12] and IEDB peptide-MHC I and II binding predictors [30,31]). These programs have several features in common: applicable to eukaryotes, can be freely downloaded, run in a standalone mode, allow high-throughput processing, and execute in a Linux environment. The emphasis here is on high-throughput. An in-house Perl script selected values (potential evidence) from the program outputs and compiled them into one file to construct the evidence profiles.

### Machine learning algorithms

Seven supervised machine learning algorithms were executed within R (a free software environment for statistical computing and graphics – http://www.r-project.org/) via R functions from packages that can be downloaded from the Comprehensive R Archive Network (CRAN): 1) decision tree, also referred to as classification and regression trees (CART) [32] via the *rpart* R function (implemented in the *rpart* package); 2) adaptive boosting [33] via the *ada* R function [34]; 3) random forest algorithm via the *randomForest* R function [35]; 4) *k*-nearest neighbour classifier (*k*-NN) via a *knn R* function [36,37] contained in the *Class* package; 5) naive Bayes classifier via a *naiveBayes R* function contained in the *e1071* package; 6) neural network (single hidden layer multilayer perceptrons) via the *nnet* R function contained in the *nnet* package [36,37]; and 7) support vector machines via the *ksvm* R function [38], which is contained in the *kernlab* package.

The algorithms were chosen because there is a wealth of literature on their successful application to a wide range of problems in multiple fields. The focus here is therefore on the application of the algorithms to solving a specific biological problem and not an evaluation or judgement of their design and logic. The application of each algorithm to building a classification model is similar in the sense that algorithm-specific R functions are used with the same training datasets. All seven machine learning R functions required at least two arguments: a data frame of categorical and/or numeric input variables (i.e. the training dataset consisting of the evidence profiles) and a class vector of ‘YES’ or ‘NO’ classification for each evidence profile i.e. target variable.

Cross-validation was performed to evaluate each training dataset and the resultant model built by each algorithm. That is, an in-house R function was used to execute the machine learning R functions multiple times (e.g. 100 runs). For each run the function randomly selected 70% of the training set to build a model. The remaining 30% of the training set was used as test data for classification. An R function called *predict*[39] was used as a generic function for predictions. An in-house Perl script summarised the multiple runs and the prediction outcomes were averaged to calculate sensitivity and specificity performance measures.

### Benchmark dataset

The benchmark dataset consisted of a collection of evidence profiles derived from *T. gondii* and *Neospora caninum* (an apicomplexan pathogen that is morphologically and developmentally similar to *T. gondii*[40]). In a similar fashion to creating the evidence profiles for the training datasets, protein sequences (140 in total) downloaded from UniProtKB were input into the seven prediction programs and an in-house Perl script compiled the evidence profiles.

It is well acknowledged in the literature that the development of vaccines directed against *T. gondii* and *N. caninum* should focus on selecting proteins that are capable of eliciting mainly a cell-mediated immune (CMI) response involving CD4 + ve T cells, Type 1 helper T cells (Th1) and Interferon-gamma (IFN-γ) in addition to a humoral response [19,41-43]. Seventy of the evidence profiles are for proteins from published studies. Twenty-two of these proteins have been observed to induce cell-mediated immune (CMI) responses and the remaining 48 have been experimentally shown to be membrane-associated or secreted. Eleven of the proteins have epitopes identified experimentally and some of these epitopes have been shown to elicit significant humoral and cellular immune responses in vaccinated mice when used in combination with other epitopes [44-47]. Additional file 1: Table S1 lists the benchmark proteins along with a publication reference to the relevant study. A brief description of the vaccine significance for some of these proteins and an entire list of evidence profiles for the benchmark dataset are also provided in Additional file 1. A further 70 evidence profiles for proteins that have been experimentally shown to be neither membrane-associated nor secreted were added to the benchmark dataset.

## Competing interests

The authors declare that they have no competing interests.

## Authors’ contributions

SG conceived and designed the experiments, performed the experiments, and analysed the data. All authors contributed to the writing of the manuscript and read and approved the final version.

## Supplementary Material

Additional file 1 **Includes typical outputs from prediction programs used for the *in silico *** **vaccine discovery pipeline, a list of the benchmark test proteins along with a publication reference to relevant studies, and a brief description of the vaccine significance for some of these proteins.** Click here for file
