# Supplementary material for: A novel strategy for classifying the output from an in silico vaccine discovery pipeline for eukaryotic pathogens using machine learning algorithms
Source: BMC Bioinformatics. 2013 Nov 2;14:315. doi: 10.1186/1471-2105-14-315 (PMC3826511; doi:10.1186/1471-2105-14-315)
Supplement: Additional file 1 — Includes typical outputs from prediction programs used for the in silico vaccine discovery pipeline, a list of the benchmark test proteins along with a publication reference to relevant studies, and a brief description of the vaccine significance for some of these proteins. [file 1471-2105-14-315-S1.pdf]

Contents

1 Introduction 1

2 Example outputs from prediction programs 1

3 Benchmark dataset..4

4 Description of protein types from Table S1 as described in recent publications. 14

4.1 Membrane-associated proteins 14

4.2 Secreted proteins 16

4.3 Miscellaneous..17

5 Epitope and MHC binding evidence...19

6 Printout of evidence profiles used in benchmark dataset......................................30

7 REFERENCES 33

1 Introduction

This document supplements the paper, ‘A novel strategy for classifying the output from an *in silico* vaccine discovery pipeline for eukaryotic pathogens using machine learning algorithms’. It is not intended to be read cover to cover but as a reference to assist the reader in a more detailed understanding of the paper, if required.

The document is in five parts: 1) Example outputs of the bioinformatics prediction programs used in the study; 2) information on the creation of the benchmark dataset including Table S1, comprising the compiled proteins with columns for Gene name, NCBI accession, UniProt ID, Protein description, Epitope experimental evidence, Organism, Study publication reference, and Comments; 3) a brief description of some of the protein types listed in Table S1 that studies have shown to be potential or at least speculative vaccine candidates; 4) Table S2 and S3, showing experimental information about epitopes and MHC binding related to proteins in Table S1; and 5) a list of ‘output values’ (i.e. evidence profiles) generated by seven prediction programs given protein sequences associated with the proteins in Table S1.

2 Example outputs from prediction programs

Selected output values from seven bioinformatics prediction programs (WoLF PSORT [1], SignalP [2], TargetP [3], TMHMM [4], Phobius [5] and IEDB peptide-MHC I and II binding predictors [6,7])) were used to test methods for vaccine candidate classification:

WoLF PSORT

Figure S1 shows a typical output from WoLF PSORT. Information about each protein sequence is displayed on separate lines (only three sequences are shown in Figure S1). Each field along the line contains a localization class (based on UniProt "Subcellular Localization" field keywords) and a score separated by a comma. There are

12 localisation classes that also map to Gene Ontology (GO)[1]. As an example of how to interpret the output in Figure S1, protein 'seq1' has six candidate sites listed in descending order of likelihood based on a score. The most likely site is extracellular (extr) and plasma membrane (plas) i.e. there is dual localisation with a score of 11.5. The plasma membrane (on its own) is the next most likely site, followed by extracellular, endoplasmic reticulum (E.R.), lysosome (lyso) and finally peroxisome (pero). The accuracy of WoLF PSORT is influenced by the number of each type of localisation site in the training data .e.g. sites with few examples in the training dataset are seldom correctly predicted.

```
seq1 extr_plas: 11.5, plas: 11, extr: 10, E.R.: 4, lyso: 4, pero: 1.5
seq2 extr: 25, lyso: 3, plas: 2, nucl: 1, E.R.: 1
seq3 extr: 31, lyso: 1
```

Figure S1. Typical output from WoLF PSORT

SignalP

It is recommended in the SignalP user manual that only the first 50 to 70 amino acids of each sequence should be used in the prediction as longer sequences increase the risk of false positives. To restrict the length of the input sequence a command-line parameter is used (e.g. –trunc 70). An example of the summary output from SignalP is shown in Figure S2. The output comprises five different scores between 0 and 1: 1) *Cmax* is the maximum "cleavage site"' score (a C-score is calculated for each position in the submitted sequence and a significant high score indicates a cleavage site); 2) *Ymax* is a derivative of the *C-score* combined with the S-score resulting in a better cleavage site prediction than the raw *C-score* alone. 3) S-max is the "maximum signal peptide" prediction score (the *S-score* for the signal peptide prediction is calculated for every single amino acid position in the submitted sequence and a high score indicates that the corresponding amino acid is part of a signal peptide, and a low score indicates that the amino acid is part of a mature protein); 4) Smean is the "average of the S-score", and 5) D is an average of the "Smean and Ymax" score. Position (pos) is the location in the amino acid sequence where Cmax (.i.e. cleavage site position), Ymax (i.e. length of signal peptide), and Smax occur. The "Y" or "N" is a yes or no indication that the sequence has a cleavage site and a signal peptide, when D is above or below the Dmaxcut. High scores also indicate that the sequence is a secretory protein. According to the authors of SignalP, a high D-score is the best indicator of secretory proteins [8].

```
# SignalP-4.0 euk predictions
# name   Cmax  pos  Ymax  pos  Smax  pos  Smean   D    ?  Dmaxcut    Networks-used
Q9UB12  0.174  31  0.302  22  0.794  14  0.660   0.495 Y  0.450      SignalP-noTM
Q58L79  0.229  49  0.234  49  0.416  48  0.193   0.218 N  0.500      SignalP-TM
Q9GU48  0.775  24  0.817  24  0.945  13  0.862   0.841 Y  0.450      SignalP-noTM
```

Figure S2. Typical summary output format from SignalP

TargetP

TargetP predicts the presence and length of secretory pathway signal peptides (SP) and mitochondrial targeting peptides (mTP) in the N-terminal presequences [9]. An example of TargetP output is shown in Figure S3. Len is the sequence length, followed by neural network scores for mitochondrial targeting peptide (mTP), secretory

[1] Gene Ontology (GO) website at: http://www.geneontology.org/

signal peptide (SP), and "other" localizations. The predicted localisation (loc) based on the scores is either mitochondrion (M) or secretory pathway (S) or any other location (-). The reliability class (RC) is from 1 (most reliable) to 5 (least reliable) and is a measure of prediction certainty. The truncated peptide length (TPlen) indicates the predicted presequence length to the cleavage site.

```
### targetp v1.1 prediction results ##########################
Number of query sequences:  3
Cleavage site predictions included.
Using OTHER networks.

Name                  Len     mTP      SP  other  Loc   RC  TPlen
----------------------------------------------------------------
Seq_1                  97   0.555  0.014  0.150   M     5   40
Seq_2                1088   0.070  0.067  0.822   _     2    -
Seq_3                 117   0.095  0.967  0.006   S     1   26
```

Figure S3. Typical output format from TargetP v1.1

TMHMM

Figure S4 shows one line of a typical output from TMHMM in a summary format. Each output line shows the length (len) of the protein sequence followed by the expected number of amino acid residues in transmembrane helices (ExpAA). If the ExpAA number is larger than 18 (a value proposed by the TMHMM creators) it is very likely to be a transmembrane protein (or have a signal peptide). The output line also shows the expected number of residues in the transmembrane helices in the first 60 amino acids of the protein (First60), the number of predicted transmembrane helices (PredHel), and the predicted protein topology i.e. the in/out orientation of the protein relative to the membrane. The creators of THHMM propose that a First60 value greater than 10 indicates a possible N-terminal signal sequence.

```
Seq_1  len=278 ExpAA=68.69 First60=39.89 PredHel=3 Topology=i7-29o44-66i87-109o
```

Figure S4. Typical summary output format from TMHMM v2.0

Phobius

Figure S5 shows the output from Phobius in a short format. The output information for one protein sequence (SEQENCE) per line consists of the number of transmembrane (TM) helices, a "Y" or "N" indicator that the sequence has a signal peptide (SP), and a predicted topology (information for only one protein sequence is shown).

```
SEQENCE  TM SP PREDICTION
Seq_1     7 Y n4-19c24/25o219-238i250-269o281-302i322-342o372-391i422-439o451-476i
```

Figure S5. Typical short output format from Phobius

T-Cell MHC class I and II binding prediction tools

Immune Epitope Database Analysis Resource (IEDB) provides a download Linux package (for a 32 bit system) that contains a collection of peptide binding prediction tools for MHC class I and class II molecules. For MHC class I the available prediction methods are: artificial neural network (ANN) [10], Average relative binding

(ARB) [11], Stabilized matrix method (SMM) [12], SMM with a Peptide-MHC Binding Energy Covariance matrix (SMMPMBEC), Scoring Matrices derived from Combinatorial Peptide Libraries (Comblib_Sidney2008) [13], Consensus [14], and NetMHCpan [15]. The available prediction methods for MHC class II are: Consensus [16], Average relative binding (ARB) [11], combinatorial library (unpublished method), NN-align [17] (this method is the equivalent to netMHCII version 2.2), SMM-align [18] (equivalent to netMHCII version 1.1), Sturniolo [19] (a method also used in the program TEPITOPE [20]), and NetMHCIIpan [21].

Figure S6 shows a typical output from the MHC class I predictor using a Consensus method (some columns have been deleted and the format adjusted to fit output on the page). Beginning at the start amino acid (numbered 1) of each sequence (denoted by #), a test subsequence of a specific peptide length (e.g. PepLengh = 9) is created (e.g. Sequence = MSMEGDRPS and is located from amino acids 1 to 9 on sequence input #1). The subsequence is scored (e.g. in units of IC_{50}nM) for binding affinity against the MHC allele e.g. HLA-A*02:05, using different prediction methods scores are calculated for each amino acid at each position in the subsequence, which are then added to yield the overall binding affinity.

Allele	#	Start	End	PepLength	Sequence	Method	IC50(nM)
HLA-A*02:05	1	1	9	9	MSMEGDRPS	NetMHCpan	6829.04
HLA-A*02:05	1	2	10	9	SMEGDRPSG	NetMHCpan	26123.53
HLA-A*02:05	1	3	11	9	MEGDRPSGA	NetMHCpan	3.32

Figure S6. Typical output from IEDB MHC I peptide binding predictor

3 Benchmark dataset

The benchmark dataset contains a compilation of *Toxoplasma gondii* and *Neospora caninum* proteins compiled from published studies that have experimentally shown the proteins to be membrane-associated or secreted. More importantly, many of the proteins were observed to induce immune responses and therefore represent the type of proteins likely to be worthwhile vaccine candidates. Eleven of the proteins have epitopes identified experimentally and some of these epitopes have been shown to elicit significant humoral and cellular immune responses in vaccinated mice when used in combination with other epitopes. The compilation of proteins is used as test data in a proof-of-concept for a classification system that is described in the paper. Two publications in particular were used to compile the protein list for the benchmark dataset. The first was a study by Rocchi and colleagues [22]. The aim was to identify tachyzoites antigens that are recognised by a cell mediated immune (CMI) response of experimentally infected animals [22]. Six *N. caninum* proteins and 16 functional orthologues of *T. gondii* were identified to elicit a CMI response. The study provided the NCBI accession numbers to these 22 identified proteins; most of which are included in Table S1 along with reference to additional studies that support Rocchi's findings. Several of the proteins are from subcellular locations other than the expected plasma membrane and extracellular sites, such as the cytoplasm (e.g. ribosomes and chaperonins), nucleus (e.g. histone H4), and enzymes (e.g. proteasome complex and glutamine synthetase). Although the latter proteins were identified in Rocchi's study to induce a CMI response, the classification system described in the paper does not classify them as potential vaccine candidates. This classification was expected as they are neither secreted nor membrane-associated, and have no epitope evidence. The assumption is that these proteins from the interior of

the pathogen are not naturally exposed to the immune system of the host but were exposed during the study as a result of the immunological procedure. Proteins that were not classified as potential vaccine candidates are indicated with 'Classification = NO' in the Comments column in Table S1. The second main study to be highlighted here is by Che and colleagues [23]. The study involved a comprehensive proteomic analysis of membrane proteins in *T. gondii*. In brief, three proteomics strategies were used: one-dimensional gel electrophoresis liquid chromatography-tandem mass spectrometry (1D gel LC-MS/MS), biotin labelling in conjunction with 1D gel LC-MS/MS analysis, and a novel strategy that combined three-layer 'Sandwich' Gel Electrophoresis (TLSGE) with multidimensional protein identification technology (MudPIT) [23]. The transmembrane protein clusters identified in the study were deposited in the Einstein Biodefense Proteomics Research Center (http://toro.aecom.yu.edu/cgi-bin/biodefense/main.cgi) and the data provided to ToxoDB (http://ToxodB.org), which is part of EuPathDB. Only proteins identified by all three strategies and having one or more predicted transmembrane segments were included in Table S1. Several proteins from the Che study in Table S1 were not classified as potential vaccine candidates by the classification system (indicated with 'Classification = NO' in the Comments column). These questionable proteins were investigated further by examining the protein's annotation in UniProt, which included links to Gene Ontology and availability of epitope evidence For the most part, the function or subcellular locations of these proteins are not annotated as membrane-associated. The annotated function or subcellular location has been included, when applicable, in the Comments column of Table S1.

It seems to be well acknowledged in the literature that the development of vaccines directed against *T. gondii* or *N. caninum* should focus on selecting proteins that are capable of eliciting mainly a CMI response involving CD4+ve T cells, Type 1 helper T cells (Th1) and Interferon-gamma (IFN-γ) (this is in addition to the humoral response) [22,24,25,26]. The types of proteins that are likely to induce the required immune response are those that are secreted from specialized organelles (micronemes, rhoptries, and dense granules). These secreted proteins are involved in the invasion and survival within host cells. The proteins typically possess a classical N-terminal signal sequence [27] for directing the protein. Following their synthesis in the cytoplasm, proteins that carry a signal peptide can be routed to no fewer than six distinct destinations: (i) plasma membrane; (ii) micronemes; (iii) apicoplast; (iv) rhoptries; (v) dense granules, and subsequently to either the parasitophorous vacuole space or the parasitophorous vacuole membrane; and (iv) inner membrane complex (IMC) [28]. The secretory proteins are likely to have secondary targeting signals responsible for precise delivery to the appropriate destination [29,30] or are delivered by a cargo receptor and chaperone protein [31]. Supposed secretory proteins without obvious signal sequences in the N-terminal are probably inaccurately annotated in UniProt, as the first exon prediction is notoriously difficult [27].

Several proteins in Table S1 that were derived from the Rocchi and Che studies are hypothetical proteins and are possibly unique to *T.gondii* or Apicomplexans in general. A BLASTP was performed using sequences of these hypothetical proteins as queries. The nearest characterised homologue protein that was found following BLASTP has been included in the Comments column when appropriate. Proteins that are used in the classification system training datasets, such as micronemal proteins (1, 4 and 6) are excluded from the test dataset.

The list of proteins in Table S1 was intended to illustrate a classification method proposed in the paper rather than to focus on any biological significance of particular vaccine candidates. The list for the purpose of a

comprehensive study of *N. caninum* and *T. gondii* vaccine candidates is acknowledged to be incomplete because an exhaustive search of the literature was not undertaken. There are some proteins in the list that have no evidence in the literature to indicate they are immunogenic or even likely to induce an immune response. These proteins do, nevertheless, have evidence that they are secreted or membrane-associated and have epitope evidence, and hence their reason for inclusion. To reiterate, the crux of the classification system is to distinguish secreted or membrane-associated proteins from all other types of proteins and especially proteins with epitope evidence. The entire premise for the *in silico* vaccine discovery approach presented in this paper is based on an *a priori* held hypothesis that a protein that is either external to or located on, or in, the membrane of a pathogen and/or contains peptides that bind to MHC molecules is more likely to be accessible to surveillance by the immune system than a protein within the interior of a pathogen [32].

The experimental evidence for the epitope and MHC binding information in Tables 2a, 2b, 3a and 3b was extracted from the Immune Epitope Database Analysis Resource (IEDB): http://www.iedb.org/.

Table S1. A list of *Toxoplasma gondii* and *Neospora caninum* proteins used in the benchmark dataset to test the classification system

Gene Name	NCBI Accession #	UniProt ID	Protein Description	Epitope evidence	Organism	Study publication reference	Comments
SAG1	AAD25091	Q9UB12	Surface antigen SAG1		*Neospora caninum*	[22,33,34,35,36]	
SAG1 (p30)	AAO72426	Q27298	SAG1 protein (P30)	YES	*Toxoplasma gondii*	[37,38,39,40,41]	53% similarity between SAG1 proteins of *T.gondii* and *N. caninum*
p35	AAD04844	O96451	Surface antigen P35		*Neospora caninum*	[34]	
p35	AAG32058	Q9GSE9	Surface antigen P35		*Toxoplasma gondii*	[23,42]	No significant similarity found between P35 of *T.gondii* and *N. caninum*
SRS2	AAX38598	Q58L79	Tachyzoite surface protein (NcSRS2)		*Neospora caninum*	[22,43,44,45,46,47]	
TGGT1_121850[2]	XP_002369822	B9PVA3	SRS domain containing protein		*Toxoplasma gondii ME49*	[22]	
GRA3	EEE24858	B6KEU8[1]	Dense granule protein 3	YES	*Toxoplasma gondii*	[23,48,49]	Contains 2 to 3 transmembrane helices + N-terminal secretory signal
NcGRA3	AAO16598	Q6YDA6	Putative dense granule protein 3		*Neospora caninum*	[48]	Homologue to B6KEU8
GRA4	AAA30142	Q27002[1]	Dense granule protein 4	YES	*Toxoplasma gondii*	[38]	Signal peptide + transmembrane helices
TGME49_086450[2]	EEB02131	B6KN48	Dense granule protein 5	YES	*Toxoplasma gondii*	[23]	
GRA7	ABF13219	A0SJB0	Granule antigen protein (GRA7)	YES	*Toxoplasma gondii*	[50,51]	Signal peptide + transmembrane helices + PTM.
TGME49_054720[2]	EEB02386	B6KNV3	Dense granule protein GRA8		*Toxoplasma gondii*	[23]	
DG1	P90661	P90661[1]	Dense granule protein 1 (NcDG1/GRA7)		*Neospora caninum*	[22,36,51]	Signal peptide + transmembrane helices + PTM. 67% similarity between GRA7 proteins of *T.gondii* and *N. caninum*
DG2	Q25540	Q25540[1]	Dense granule protein 2		*Neospora caninum*	[52]	2 transmembrane helices + PTM.

Abbreviation: PTM = post-translational modification
[1] Protein manually annotated and reviewed in UniProtKB. All other proteins are automatically annotated and not reviewed in UniProtK.
[2] ORF name – a name temporarily attributed to an open reading frame (ORF) by a sequencing project.

Gene Name	NCBI Accession #	UniProt ID	Protein Description	Epitope evidence	Organism	Study publication reference	Comments
GRA2	AAG28489	Q9GU48	GRA2 protein		*Neospora caninum*	[22,53,54]	No significant similarity found between proteins Q25540 and Q9GU48
ROP1	AAA69859	Q04151	Rhoptry protein	YES	*Toxoplasma gondii*	[55,56]	Signal peptide
	XP_002370897	Q06AK3	Rhoptry antigen, putative (ROP2)		*Toxoplasma gondii ME49*	[22,38,41,57]	
ROP4	ABU24469	A7UDC8	Secretory rhoptry 4	YES	*Toxoplasma gondii*	[38,58]	
TGME49_109590[2]	EEE32684	B6KA38	Surface protein rhoptry, putative	YES	*Toxoplasma gondii VEG*	[22]	
TGME49_015780[2]	XP_002370897	B6KSS4	Rhoptry antigen, putative (ROP8)		*Toxoplasma gondii ME49*	[23,59]	
ROP18	EEB00617	B6KIB2	Rhoptry kinase family protein		*Toxoplasma gondii*	[23,60,61,62]	
TGME49_052360[2]	EEB02204	B6KNC1	Rhoptry protein, putative		*Toxoplasma gondii*	[23]	
RON1	AAZ38162	Q45WA9	Rhoptry neck protein 1		*Toxoplasma gondii*	[23,63,64]	
RON2	EEB04593	B6KV60	Rhoptry neck protein 2		*Toxoplasma gondii*	[23,65]	
RON3	AAZ38164	Q45WA7	Rhoptry neck protein 3		*Toxoplasma gondii*	[23,63,64]	
	ACK57540	B7UDF2	Rhoptry neck protein 8		*Toxoplasma gondii*	[23,63,64]	
Nc-Mic3	AAF19184	Q9U483	Microneme protein Nc-P38 (Nc-Mic3)		*Neospora caninum*	[22,57,66]	
MIC3	CAB56644	Q9GRG4	MIC3 microneme protein	YES	*Toxoplasma gondii*	[67,68]	Signal peptide
MIC11	AAN16380	Q8IT72	Microneme protein NcMIC11 precursor		*Neospora caninum*	[22,69]	
MIC13	AFD54629	B0LUH4	Microneme protein 13		*Toxoplasma gondii*	[70]	Signal peptide
TGME49_058950[2]	EEA97968.	B6KBC5	Lectin-domain protein		*Toxoplasma gondii*	[23,71]	*T.gondii* MIC1 is a lectin [72]
TGME49_110000[2]	EEA97105	B6KA68	Lung seven transmembrane receptor domain-containing protein		*Toxoplasma gondii*	[23]	

Abbreviation: PTM = post-translational modification
[1] Protein manually annotated and reviewed in UniProtKB. All other proteins are automatically annotated and not reviewed in UniProtK.
[2] ORF name – a name temporarily attributed to an open reading frame (ORF) by a sequencing project.

Gene Name	NCBI Accession #	UniProt ID	Protein Description	Epitope evidence	Organism	Study publication reference	Comments
TGME49_099110[2]	EEB04639	B6KVA6	Cleft lip and palate transmembrane protein 1		*Toxoplasma gondii*	[23]	32% similarity between *T.gondii* and *H. sapiens* proteins
TGME49_005240[2]	EEB00616	B6KIB1	Cleft lip and palate associated transmembrane protein 1, putative		*Toxoplasma gondii*	[23]	33% similarity between B6KVA6 and B6KIB1
TGME49_036020[2]	EEB01847	B6KMB4	BT1 transmembrane domain-containing protein		*Toxoplasma gondii*	[23,73]	
GT1	EEB03625	Q8MUM2	Facilitative glucose transporter		*Toxoplasma gondii*	[23,74,75]	
TGME49_030420[2]	EEB00813	B6KJD0	Calcium-transporting ATPase		*Toxoplasma gondii*	[23,76]	
GAP45	Q7Z289	Q7Z289	Gliding-associated protein 45		*Toxoplasma gondii*	[23,77]	
GAP50	EEB03556	Q6PQ42	Membrane anchor for myosin XIV		*Toxoplasma gondii*	[23,78]	Signal peptide
	P84343	P84343[1]	Peptidyl-prolyl cis-trans isomerase (Belongs to the cyclophilin-type PPIase family)		*Neospora caninum*	[26]	Signal peptide
TGME49_109560[2]	EEA97072	B6KA35	Nmda receptor glutamate-binding chain		*Toxoplasma gondii*	[23]	
TGGT1_123090[2]	EEB02615	B6KPR6	Major sperm protein domain-containing protein		*Toxoplasma gondii*	[23]	
TGME49_026430[2]	EEA99155	B6KEM3	Reticulon domain-containing protein		*Toxoplasma gondii*	[23]	
TGGT1_123170[2]	EEB04156	B6KTX3	TB2/DP1, HVA22 domain-containing protein		*Toxoplasma gondii*	[23]	
TGME49_032830[2]	EEB01012	B6KJX9	Vacuolar proton-translocating ATPase subunit		*Toxoplasma gondii*	[23]	Vacuolar proton-translocating ATPases (V-ATPases) are responsible for organelle acidification in all eukaryotic cells [79]
TGME49_058830[2]	EEA97956	B6KBB3	Suppressor of actin mutations 2/vacuolar sorting protein, putative		*Toxoplasma gondii*	[23]	Actin is multi-functional protein found in all eukaryotic cells. Vacuolar sorting protein in Yeast is an ATPase required for endosomal trafficking [80]

Abbreviation: PTM = post-translational modification
[1] Protein manually annotated and reviewed in UniProtKB. All other proteins are automatically annotated and not reviewed in UniProtK.
[2] ORF name – a name temporarily attributed to an open reading frame (ORF) by a sequencing project.

Gene Name	NCBI Accession #	UniProt ID	Protein Description	Epitope evidence	Organism	Study publication reference	Comments
TGGT1_104500[2]	EEE23454	B9PRX5	Proteasome subunit alpha type, putative		*Toxoplasma gondii GT1*	[22]	Possible false positive as the protein is located in nucleus and cytoplasm
TGVEG_051970[2]	EEE30125	B9QH60	Acetyl-CoA carboxylase, putative		*Toxoplasma gondii VEG*	[22,81,82]	
TGME49_073490[2]	XP_002365950	B6KDM7	Glutamine synthetase, putative		*Toxoplasma gondii ME49*	[22]	Possible false positive as the protein is located in cytoplasm
TGME49_090160[2]	XP_002368431	B6KKQ8	Sortilin, putative		*Toxoplasma gondii*	[23,83]	
TGME49_055260[2]	EEA97713	B6KAM0	Apical membrane antigen 1, putative		*Toxoplasma gondii*	[23,84,85,86]	
TGME49_074060[2]	EEA98866	B6KDT4	Mitochondrial 2-oxoglutarate/malate carrier protein, putative		*Toxoplasma gondii*	[23]	Catalyzes the transport of 2-oxoglutarate across inner mitochondrial membrane in an exchange for malate [UniProt description]
TGGT1_103360[2]	EEB03462	B9PRN1	Phosphate carrier protein, putative		*Toxoplasma gondii*	[23]	Transport of phosphate groups from the cytosol to the mitochondrial matrix [UniProt description]
TGGT1_101590[2]	EEE23237	B9PRA8	ADP/ATP carrier, putative		*Toxoplasma gondii*	[23]	Mitochondrial carrier
TGGT1_025650[2]	EEE23643	B9PPS0	Thioredoxin, putative		*Toxoplasma gondii*	[23,87]	
TGGT1_095290[2]	EEE19397	B9Q2T7	Conserved hypothetical protein		*Toxoplasma gondii*	[23]	Homologous to RON2
TGGT1_117620[2]	EEE27018	B9PHB6	Conserved hypothetical protein		*Toxoplasma gondii*	[23]	Nearest characterised homologue protein from *Drosophila melanogaster* (25%)
TGGT1_031220[2]	EEE22451	B9PT54	Conserved hypothetical protein		*Toxoplasma gondii GT1*	[22]	Nearest homologue characterised protein from *Candida lipolytica* (27%)

Abbreviation: PTM = post-translational modification
[1] Protein manually annotated and reviewed in UniProtKB. All other proteins are automatically annotated and not reviewed in UniProtK.
[2] ORF name – a name temporarily attributed to an open reading frame (ORF) by a sequencing project.

Gene Name	NCBI Accession #	UniProt ID	Protein Description	Epitope evidence	Organism	Study publication reference	Comments
TGGT1_039470[2]	EEE23072	B9PSP5	Conserved hypothetical protein		*Toxoplasma gondii GT1*	[22]	Nearest characterised homologue protein is SRS domain-containing protein from *N. caninum* (35%)
TGME49_047770[2]	EEE31815	B6KH21	Conserved hypothetical protein		*Toxoplasma gondii*	[23]	No characterised homologue protein,
TGME49_079100[2]	EEB04961	B6KW78	Hypothetical protein		*Toxoplasma gondii*	[23]	Nearest characterised homologue protein from *Alcaligenes faecalis* (28%)
TGME49_005740[2]	EEB00665	B6KIG0	Hypothetical protein		*Toxoplasma gondii*	[23]	Nearest characterised homologue protein from *Ricinus communis* (26%)
TGME49_058870[2]	EEA97960	B6KBB7	Hypothetical protein		*Toxoplasma gondii*	[23]	Nearest characterised homologue protein from *Pleurage anserina* (29%)
TGME49_020950[2]	EEB02805	B6KQ22	Hypothetical protein		*Toxoplasma gondii*	[23]	Nearest characterised homologue protein from *Anopheles gambiae* (51%)
TGME49_012300[2]	EEB04691	B6KVF8	Hypothetical protein	YES	*Toxoplasma gondii*	[23]	Nearest characterised homologue protein from *Coprinopsis cinerea* (23%)
TGME49_062610[2]	EEA98220	B6KC27	Hypothetical protein		*Toxoplasma gondii*	[23]	Nearest characterised homologue protein is Serpentine receptor from *Plasmodium falciparum* (26%)
TGME49_038130[2]	EEA99364	B6KGA5	Hypothetical protein		*Toxoplasma gondii*	[23]	Nearest characterised homologue protein from *Cellvibrio sp.* (38%)
TGME49_025850[2]	EEA99107	B6KEH5	Hypothetical protein		*Toxoplasma gondii*	[23]	Nearest characterised homologue protein is Fxna (putative) from *T. gondii* (99%)
TGME49_034410[2]	EEB01741	B6KM08	Hypothetical protein		*Toxoplasma gondii*	[23]	Nearest characterised homologue protein is Mechanosensitive ion channel from *Plasmodium falciparum* (48%)

Abbreviation: PTM = post-translational modification
[1] Protein manually annotated and reviewed in UniProtKB. All other proteins are automatically annotated and not reviewed in UniProtK.
[2] ORF name – a name temporarily attributed to an open reading frame (ORF) by a sequencing project.

Gene Name	NCBI Accession #	UniProt ID	Protein Description	Epitope evidence	Organism	Study publication reference	Comments
TGGT1_121070[2]	EEB02749	B6KPL7	Ubiquinol-cytochrome C reductase iron-sulfur subunit, putative		*Toxoplasma gondii*	[23]	Classification = NO. Possible role: transmembrane protein
TGME49_056030[2]	EEA97769	B6KAS6	Tubulin polymerization promoting protein		*Toxoplasma gondii*	[23]	Classification = NO. Possible role: intracellular signal transduction
TGME49_046530[2]	EEE31919	B6KGR8	Phosphatidylglycerophosphate synthase, putative		*Toxoplasma gondii*	[23]	Classification = NO. Possible role: phospholipid biosynthetic process
TGME49_063060[2]	EEA98265	B6KC72	26S proteasome non-ATPase regulatory subunit 1, putative		*Toxoplasma gondii*	[23]	Classification = NO. Possible role: regulation of catalytic activity and part of the proteasome
TGME49_110760[2]	EEA97181	B6KAE4	Protein phosphatase 2C, putative		*Toxoplasma gondii*	[23]	NO classification. Possible role: protein dephosphorylation
TGME49_049990[2]	EEE31624	B6KHK8	Microtubule-binding protein, putative		*Toxoplasma gondii*	[23]	Classification = NO. Possible role: interact with the microtubules of the cellular cytoskeleton
TGME49_115770[2]	EEA97614	B6K9N1	Cytochrome P450, putative		*Toxoplasma gondii*	[23]	Classification = NO. Possible role: enzyme that catalyses the oxidation of organic substances
TGME49_090950[2]	XP_002368479	B6KKV6	Clathrin heavy chain, putative		*Toxoplasma gondii*	[23]	Classification = NO. Possible role: intracellular protein transport
TGME49_049840[2]	EEE31638	B6KHJ3	Ciliary dynein heavy chain, putative		*Toxoplasma gondii*	[23]	Classification = NO. Possible role: ATP catabolic process
TGGT1_051710[2]	EEE29336	B9PNK6	Histone H4, putative		*Toxoplasma gondii VEG*	[22]	Classification = NO. Subcellular location: nucleus
	AAD38419	Q9Y1U8	60 kDa chaperonin 2		*Toxoplasma gondii*	[22]	Classification = NO. Subcellular location: cytoplasm

Abbreviation: PTM = post-translational modification
[1] Protein manually annotated and reviewed in UniProtKB. All other proteins are automatically annotated and not reviewed in UniProtK.
[2] ORF name – a name temporarily attributed to an open reading frame (ORF) by a sequencing project.

Gene Name	NCBI Accession #	UniProt ID	Protein Description	Epitope evidence	Organism	Study publication reference	Comments
TGGT1_098790[2]	EEE20214	B9Q0G6	20S proteasome subunit alpha, putative		*Toxoplasma gondii GT1*	[22]	Classification = NO. Subcellular location: cytoplasm, nucleus
TGGT1_027600[2]	EEE23774	B9PQ51	40S ribosomal protein S8, putative		*Toxoplasma gondii GT1*	[22]	Classification = NO. Subcellular location: cytoplasm, nucleus
TGME49_039500[2]	XP_002366589	B9PNI2	Proteasome subunit alpha type 4, subunit		*Toxoplasma gondii ME49*	[22]	Classification = NO. Subcellular location: cytoplasm, nucleus
TGGT1_055480[2]	EEE19215	B9Q3C3	Proteasome subunit beta type, putative		*Toxoplasma gondii GT1*	[22]	Classification = NO. Subcellular location: cytoplasm, nucleus
TGGT1_012060[2]	EEE25357	B9PL62	Proteasome subunit alpha type, putative		*Toxoplasma gondii GT1*	[22]	Classification = NO. Subcellular location: cytoplasm, nucleus
TGME49_087210[2]	XP_002369317	B9PSG2	Proteasome subunit alpha type 2, putative		*Toxoplasma gondii ME49*	[22]	Classification = NO. Subcellular location: cytoplasm, nucleus
TGME49_009680[2]	EEB02577	B6KPE4	Hypothetical protein		*Toxoplasma gondii*	[23]	Classification = NO. Nearest characterised homologue protein is Nucleolar GTPase/ATPase p130 from *Ostreococcus tauri* (34%)
TGME49_118140[2]	EEB02613	B6KPR4	Hypothetical protein		*Toxoplasma gondii*	[23]	Classification = NO. Nearest characterised homologue protein is U4/U6.U5 tri-snRNP-associated from *Mus musculus* (26%) Possible role: mRNA splicing in nucleus
TGME49_062650[2]	EEA98224	B6KC31	Hypothetical protein		*Toxoplasma gondii*	[23]	Classification = NO. Nearest characterised homologue protein is WD40 repeat-like from *Neurospora tetrasperma* (26%) Possible role: RNA processing

Abbreviation: PTM = post-translational modification
[1] Protein manually annotated and reviewed in UniProtKB. All other proteins are automatically annotated and not reviewed in UniProtK.
[2] ORF name – a name temporarily attributed to an open reading frame (ORF) by a sequencing project.

4 Description of protein types from Table S1 as described in recent publications.

The following proteins are in no particular order of importance but are grouped into three sections: membrane-associated, secreted, and miscellaneous.

4.1 Membrane-associated proteins

SRS2 (or Nc-p43) [88] is localised on the surface of *N. caninum* of both bradyzoites and tachyzoites [89]. Collectively, surface antigens are known as the SRS (SAG1-related sequences) superfamily of proteins. The SRS2 protein is involved in the host cell invasion process [90] and polyclonal and monoclonal antibodies directed against it were shown to inhibit invasion of placental ovine trophoblasts *in vitro* [91].

Several rodent studies using NcSR2 as a vaccine against *N. caninum* tachyzoites have demonstrated improved survival for the host [44], a Th2 immune response with reduced transplacental transmission [45], and humoral and cellular immune responses [46]. In two cattle studies, vaccines incorporating NcSRS2 induced T-lymphocyte activation and IFN-γ secretion [43,47].

SAG1 is a tachyzoite glycosylphosphatidylinositol (GPI)-anchored surface molecule [92] involved in host cell attachment and invasion [93] and is antigenically immunodominant [34].

Two surface proteins of 29 and 35 kDa (designated Ncp29/NcSAG1 and Ncp35/NcSRS2, respectively) from *N. caninum* tachyzoites were identified [34]. Localization studies and surface labelling with biotin demonstrated that Ncp29 and Ncp35 are membrane-associated and displayed on the surface of the parasite. Ncp29 and Ncp35 were characterised as GPI-anchored surface proteins. Sequence comparisons of Ncp29 and Ncp35 with GenBank sequences indicated that they are most similar to the *T. gondii* surface antigen 1 (SAG1) and surface antigen 1-related sequence 2 (SRS2), respectively. Consequently, Ncp29 has been designated NcSAG1 and Ncp35 has been designated NcSRS2. Both NcSAG1 and NcSRS2 contain a tandem duplicated motif and 12 conserved cysteines, which are also found in all of the SAG and SRS proteins of *T. gondii* [34].

Recombinant vaccinia viruses expressing the surface protein of NcSAG1 (or NcSRS2) were constructed and shown to effectively protect from an *N. caninum* invasion in a mouse model system (the efficacy of NcSRS2 was higher than that of NcSAG1) [36]. In other studies, mice immunized with r-SAG1 delayed death for 60 hours when challenged with *T gondii* RH tachyzoites [35]; a combined DNA/recombinant antigen-vaccine, based on NcSAG1 and NcSRS2, respectively, exhibited a highly significant protective effect against experimentally induced cerebral neosporosis in mice [33]; and a combined vaccination with NcSRS2 and NcDG1 showed protective effects against experimental infection in gerbils [44].

Using liposomes as adjuvant, a purified membrane antigen from *T. gondii* (SAG1 p30) was shown to provide protection of mice from a fatal *T. gondii* infection [37]. In another study, immune splenocytes from mice immunized with p30 appeared to lyse peritoneal macrophages infected with *T. gondii* [40].

SRS domain containing proteins (XP_002369822) are present in large numbers on the parasite surface and facilitate the invasion of multiple host and cell types [94]. They are considered to be extremely immunogenic in *Toxoplasma* [22].

Apical membrane antigen 1 (AMA1) is a conserved transmembrane adhesin of apicomplexan parasites and is an essential component of the moving junction complex involved in host-cell invasion. *T. gondii* AMA1 is secreted onto the parasite surface and subsequently released by proteolytic cleavage within its transmembrane domain [85]. The *Plasmodium* apical membrane antigen 1 has been shown to elicit a protective immune response against merozoites dependent on the correct pairing of its numerous disulfide bonds [84]. In a study using preincubation of free tachyzoites with anti-rNcAMA1 (a *N. caninum* AMA1 recombinant), IgG antibodies inhibited the invasion into host cells by *N. caninum* and *T gondii* [86]. The latter indicates a potential common vaccine candidate to control two parasites.

RON1/RON2/RON3/RON8 are proteins originating from the neck of the rhoptries. All RON proteins have been demonstrated to be present at the moving junction between the apex of Apicomplexa and the host cell membrane that moves along the parasite and serves as support to propel it inside the host cell [63]. The moving junction assembly is initiated by injection of RONs into the host cell, where RON2 spans the membrane and functions as a receptor for apical membrane antigen 1 (AMA1) on the parasite [65]. There is no evidence in the literature to indicate that RONs are immunogenic. RONs were included in the test dataset because the proteomics Che study included them as members of transmembrane proteins. Interestingly, the five prediction programs typically indicate RONs as both membrane-associated and secreted.

Biopterin transport (BT) Transmembrane protein: Massimine and colleagues report that the presence of putative folate transporter genes in the *Toxoplasma* genome, which are homologous to the BT1 family of proteins, suggests that *Toxoplasma* may encode proteins involved in folate transport. Folates are key elements in eukaryotic biosynthetic processes. In a study [95], BT1 in the species ***Leishmania donovani*** was inactivated by gene disruption mediated by homologous recombination. The ***L. donovani BT1*** null mutant (i.e. an attenuated organism) showed less capacity to induce infection in mice than wild-type parasites and could elicit protective immunity in mice susceptible to infection against a ***L. donovani*** challenge [95]. The folate transport mechanism therefore represents a novel target in a vaccination strategy or the development of new drugs [73].

Calcium-transporting ATPase: Calcium controls a number of vital processes in apicomplexans including protein secretion, motility, and differentiation [76]. ATPases are membrane-bound transporters that couple ion movement through a membrane with the synthesis or hydrolysis of a nucleotide, usually ATP. A study showed evidence of a *T. gondii* plasma membrane-type Ca2+ ATPases and suggested that parasite calcium pathways may be exploited as new therapeutic targets for intervention [76]. The process of invasion involves two Ca2+ dependent events: protrusion of the conoid and the induced secretion of adhesive complexes from the micronemes [28]. Immunolocalization and challenge studies using a recombinant *Vibrio cholerae* ghost expressing *Trypanosoma brucei* Ca2+ ATPase (TBCA2) antigen demonstrated immune responses in mice [96]. However, the immunization failed to protect the mice against a *T. brucei* challenge, despite the inducement of antigen-specific antibodies, Th1 cytokines, interleukin-2, and IFN-γ,

Glucose transporter protein: *T. gondii* uses host sugars for energy and to generate glycoconjugates that are important to its survival and virulence. A glucose transporter protein facilitates in transporting mannose, galactose, fructose, glucose, and hexose at its plasma membrane. A study [74] demonstrates that glucose is nonessential for *T. gondii* tachyzoites. However, a study has validated a hexose transporter of *Plasmodium falciparum* as a novel drug target [97]. There is no literature implicating transporter proteins as vaccine candidates. Segments of transporter proteins are nevertheless exposed to the immune system.

4.2 Secreted proteins

GRA2/GRA3/GRA4/GRA7 are dense granule proteins involved in the cellular invasion process. Dense granules are secretory vesicles that play a major role in the structural modifications of the parasitophorous vacuole (PV) in which the parasite develops [53].

Escherichia coli expressed NcGRA2 demonstrated immunogenicity in an immunization/challenge mouse model of transplacental transmission, but only partial reduction against foetal infection and pup mortality [98]. Similarly in another study, vaccination of mice with recombinant NcGRA2 expressed in a *Brucella abortus* strain induced only partial protection against transplacental transmission with a mortality of 10-50% [54].

Immunization of mice with plasmid DNA expressing NcGRA7 conferred partial protection against congenital neosporosis [51]. Also, both humoral and cellular immune responses against *T. gondii* was detected in sheep immunized with DNA plasmids encoding *T. gondii* GRA7 formulated in an adjuvant formulation [50].

Studies using antibodies to immunolocalize the *T. gondii* dense granule protein GRA3 have shown that this protein associates strongly with the parasitophorous vacuole membrane (PVM) i.e. GRA3 has an N-terminal secretory signal sequence and a transmembrane domain consistent with its insertion into the PVM. A homologue was identified in *N. caninum* (UniProtID Q6YDA6). GRA3 possesses a dilysine 'KKXX' endoplasmic reticulum (ER) retrieval motif that interacts with PVM and the calcium modulating ligand of host cell ER in the parasitism of *T. gondii* [48,49]. There is no evidence in the literature that GRA3 induces an immune response. However, the findings on GRA3 support the fact that the five prediction programs indicate that GRA3, and most other dense granule proteins described here, are both membrane-associated and secreted. GRA2 and GRA4 are not predicted to be membrane-associated.

NcMIC11/ Nc-Mic3/ MIC3 are from micronemes, which are secretory organelles, and are discharged by exocytosis during the attachment to the host cell surface to facilitate cell invasion [99]. Many microneme proteins also contain well-conserved functional domains associated with mainly adhesive activity (e.g. EGF-like and PAN_1 domains) and some protease activity (e.g. Peptidase_S8 and Rhomboid) [27].

MIC3 is expressed in all three infectious stages of *T. gondii* (tachyzoites, bradyzoites, and sporozoites). A DNA vaccine encoding the MIC3 protein has been demonstrated to elicit a strong specific immune response providing significant protection against *T. gondii* infection [68].

ROP1/ROP2/ROP4/ROP18 are secreted proteins from rhoptries (specialized secretory organelles in the apical complex) and are involved in a variety of cellular functions related to host cell invasion, formation of the parasitophorous vacuole, and parasite-host cell interplay [100].

The protein combinations of rROP2 + rROP4 + rGRA4 and rROP2 + rROP4 + rSAG1 were shown to be very effective in the development of a high level of protection irrespective of the genetic backgrounds and innate resistance to toxoplasmosis of the laboratory mice [38].

A DNA vaccine encoding the ROP1 antigen of *T.gondii* and ovine CD154 was demonstrated to stimulate humoral and cellular immune responses in sheep. The intramuscular injection of pROP1 only induced a Th1-specific immune response [55].

Vaccination with recombinant NcROP2 induces a protective Th-1-biased or Th-2-biased immune response against experimental *N. caninum* in mice (depends also on the adjuvant used) [100]; fusion proteins ROP2-SAG1 exhibit immunogenicity as a recombinant protein vaccine, or DNA vaccine, or DNA boosted with protein

immunization procedure [41]; and NcMIC1, NcMIC3, and NcROP2 applied either as single vaccines or as vaccine combinations leads to a significant protection against vertical transmission of *N. caninum* in mice [57].

The polymorphic rhoptry protein kinase ROP18 was recently shown to determine the difference in virulence between the *T. gondii* types I, II and III strains (which are prevalent in North America and Europe) by phosphorylating and inactivating the IFN-γ-induced immunity-related Guanosine Triphosphatases (IRGs) that promote killing by disrupting the parasitophorous vacuole membrane (PVM) in murine cells [62].

Cyclophilins (Peptidyl-prolyl cis-trans isomerase) are ubiquitous cytosolic proteins. A study has demonstrated cyclophilin (NcCyP) is present in *N. caninum* tachyzoites and is a major component responsible for the induction of IFN-γ production [26]. The production of IFN-γ in response to intracellular microbial exposure is critical to the development of a host protective immunity to control the acute phase of neosporosis. [101]. NcCyP is a secretory protein.

4.3 Miscellaneous

The following proteins were included in the test dataset because the proteomics Che study identified them as transmembrane proteins. There is no evidence in the literature that these proteins induce an immune response and, from their annotated descriptions, are unlikely vaccine candidates i.e. they are not associated with the plasma membrane. The proteins remain in the test dataset essentially because proteins of these types are expected to be classified as vaccine candidates in a deployment of the classification system i.e. the prediction programs *predict* that they are membrane-associated given their protein sequences. Whether these proteins, or in fact any classified candidate, prove to be false positives, can only be determined in the laboratory.

Sortilin-like receptor is a transmembrane cargo receptor that functions in transport to the endolysosome system in yeast and mammals [31]. *T. gondii* sortilin-like receptor is required for the subcellular localization and formation of apical secretory organelles. It is a transmembrane protein that resides within Golgi-endosomal related compartments. The lumenal domain specifically interacts with rhoptry and microneme proteins, while the cytoplasmic tail recruits cytosolic sorting machinery involved in anterograde and retrograde protein transport [83]

Gliding-associated proteins (GAPs) are components of the glideosome. The glideosome is a unique attribute of the Apicomplexa phylum and is an actin- and myosin-based machine [77]. This macromolecular machine provides the gliding motility for parasite migration across biological barriers and for host-cell invasion and egress [28]. The glideosome is assumed not to be exposed to the immune system as it is located between the plasma membrane and inner membrane complex (IMC). GAP45 is anchored to the plasma membrane and IMC via its N- and C-terminal extremities, respectively.

Acetyl-CoA carboxylase (ACC) is an enzyme involved in fatty acid synthesis. This enzyme is synthesized in the cytosol and transported into the apicoplast [81]. Aryloxyphenoxypropionates, inhibitors of the plastid acetyl-CoA carboxylase (ACC) of grasses, also inhibit *T. gondii* ACC [82].

Thioredoxin protein: The apicoplast in *T.gondii* is an essential chloroplast-related organelle, bounded by multiple membranes, to which proteins are trafficked via the secretory system. The thioredoxin protein in *T. gondii* is apicoplast-associated, which is predominantly soluble or peripherally associated with membranes, and which localizes primarily to the outer compartments of the apicoplast [87]. Research is investigating a role for

the apicoplast in vaccine strategies. Genetically attenuated malaria parasites (with deleted genes that encode for apicoplast fatty acid biosynthesis) have been trialled and provide sterile immunity in mice for 210 days [102]. Apicoplast fatty acid biosynthesis is essential for organelle biogenesis and parasite survival in *T. gondii* hosted by mice [103].

Lectin-domain protein: *T. gondii* has as broad host cell specificity suggesting that adhesion should involve the recognition of ubiquitous surface-exposed host molecules or, alternatively, the presence of various parasite attachment molecules able to recognize different host cell receptors [71]. In a study [71], a sugar-binding activity (lectin) in tachyzoites of *T. gondii* was discovered that plays a role *in vitro* in erythrocyte agglutination and infection of human fibroblasts and epithelial cells. The results of the study suggest that the attachment of *T. gondii* to its target cell is mediated by parasite lectins and that sulfated sugars on the surface of host cells may function as a key parasite receptor.

5 Epitope and MHC binding evidence

Table S2a. Experimentally validated epitopes related to benchmark proteins in Table S1

UniProt ID	IEDB Epitope ID	Sub-sequence	NCBI GI	Source Molecule	PubMed ID
Q27298	19583	GFLTSMFPK	37778533	SAG1 protein	10569750
Q27298	40286	LVCGKDGVK	37778533	SAG1 protein	10569750
Q27298	60031	SPEKHHCTV	37778533	SAG1 protein	10569750
Q27298	61463	SSVVNNVAR	37778533	SAG1 protein	10569750
Q27298	65118	TLVCGKDGV	37778533	SAG1 protein	10569750
Q27298	71330	VTGLIGSI	37778533	SAG1 protein	10569750
Q27298	140697	KSFKDILPK	37778533	SAG1 protein	20347630
B6KEU8	167481	ADQPGNHQALAEPV	237834147	dense granule protein 3	22470537
Q27002	30927	KGFGGTRTSTAPAEAGKTELDDGYRPPPFNPRPSPYAELLKDLERMRKE	2498423	Dense granule protein 4 precursor	9618729
Q27002	58128	SGLTGVKDSSS	2498423	Dense granule protein 4 precursor	18555564
Q27002	104255	SPMNGGYYM	2498423	Dense granule protein 4 precursor	
Q27002	156546	STEDSGLTGVKDSSS	2498423	Dense granule protein 4 precursor	21939715

UniProt ID	IEDB Epitope ID	Sub-sequence	NCBI GI	Source Molecule	PubMed ID
Q27002	167686	TEDSGLTGVKDSSS	2498423	Dense granule protein 4 precursor	22470537
Q27002	167910	GGTRTSTAPAEAGKTE	2498423	Dense granule protein 4 precursor	22496494
Q27002	167956	LDDGYRPPPFNPRPSP	2498423	Dense granule protein 4 precursor	22496494
Q27002	167983	PAEAGKTELDDGYRPP	2498423	Dense granule protein 4 precursor	22496494
Q27002	167991	PPPFNPRPSPYAELLK	2498423	Dense granule protein 4 precursor	22496494
Q04151	167628	NSEDDDTFHDA	897823	rhoptry protein	22470537
Q04151	167648	PVRGPDQVPA	897823	rhoptry protein	22470537
B6KA38	167629	NSEDDTFHDA	237829837	rhoptry protein, putative	22470537
B6KA38	167647	PVRDPRQVPGRGE	237829837	rhoptry protein, putative	22470537
B6KA38	167651	QELPPPNAQEL	237829837	rhoptry protein, putative	22470537
B6KA38	167691	TRVRGALRGRGR	237829837	rhoptry protein, putative	22470537
Q9GRG4	167868	ALPIQKSVQLGSFDKV	5931754	MIC3 microneme protein	22496494
Q9GRG4	167875	CEKEFGISASSCKCDN	5931754	MIC3 microneme protein	22496494
Q9GRG4	167885	DKVVPSREVVSESLAP	5931754	MIC3 microneme protein	22496494
Q9GRG4	167907	GETLVNLPEGGQGCKR	5931754	MIC3 microneme protein	22496494

UniProt ID	IEDB Epitope ID	Sub-sequence	NCBI GI	Source Molecule	PubMed ID
Q9GRG4	167917	GSEGSLSEKMNIVFKC	5931754	MIC3 microneme protein	22496494
Q9GRG4	167921	GVEVTLAEKCEKEFGI	5931754	MIC3 microneme protein	22496494
Q9GRG4	167923	HAFRENCSPGRCIDDA	5931754	MIC3 microneme protein	22496494
Q9GRG4	167963	LLHALTFSGAVWMCTP	5931754	MIC3 microneme protein	22496494
Q9GRG4	167998	RQLHTDNGYFIGASCP	5931754	MIC3 microneme protein	22496494
Q9GRG4	168006	SKRGNAKCGPNGTCIV	5931754	MIC3 microneme protein	22496494

Table S2b. Experimentally validated epitopes related to homologues of proteins in Table S1

UniProt ID	Homologue UniProt ID	IEDB Epitope ID	Sub-sequence	NCBI GI	Source Molecule	PubMed ID
Q27298	P13664	1161	AESKSVII	129348	Major surface antigen p30 precursor	10569750
Q27298	P13664	6688	CNEKSFKDILPKLTENPWQ	129348	Major surface antigen p30 precursor	7997247
Q27298	P13664	6784	CPKTALTEPPTLAYSPNRQIC	129348	Major surface antigen p30 precursor	7997247
Q27298	P13664	21822	GPVKLSAEGPTTMTLV	129348	Major surface antigen p30 precursor	
Q27298	P13664	27248	ILPKLTENPWQ	129348	Major surface antigen p30 precursor	18555564

UniProt ID	Homologue UniProt ID	IEDB Epitope ID	Sub-sequence	NCBI GI	Source Molecule	PubMed ID
Q27298	P13664	33548	KSVIIGCTGGSPEKHHC	129348	Major surface antigen p30 precursor	7997247
Q27298	P13664	40287	LVCGKDGVKVPQDNNQYC	129348	Major surface antigen p30 precursor	7997247
Q27298	P13664	43659	NEKSFKDI	129348	Major surface antigen p30 precursor	10569750
Q27298	P13664	45276	NNVARCSYGADSTLGPV	129348	Major surface antigen p30 precursor	11578086
Q27298	P13664	52512	QTFVVGCI	129348	Major surface antigen p30 precursor	10569750
Q27298	P13664	57259	SDPPLVANQVVTCPDKKSTA	129348	Major surface antigen p30 precursor	7997247
Q27298	P13664	63089	TCPDKKSTA	129348	Major surface antigen p30 precursor	21939715
Q27298	P13664	65781	TPTENHFTL	129348	Major surface antigen p30 precursor	
Q27298	P13664	71566	VTVTVQARASSVVNNV	129348	Major surface antigen p30 precursor	

UniProt ID	Homologue UniProt ID	IEDB Epitope ID	Sub-sequence	NCBI GI	Source Molecule	PubMed ID
Q27298	P13664	71567	VTVTVQARASSVVNNVARCSYGADSTLGPVKLSAEGPTTMT	129348	Major surface antigen p30 precursor	
Q27298	P13664	167860	AFPAESKSVIIGCTGGSPE	129348	Major surface antigen p30 precursor	22496494
Q27298	P13664	167861	AGAAGSAKSAAGTASHVSI	129348	Major surface antigen p30 precursor	22496494
Q27298	P13664	167862	AGTTSSCTSKAVTLSSLIP	129348	Major surface antigen p30 precursor	22496494
Q27298	P13664	167872	ASHVSIFAMVIGLIGSIAACVA	129348	Major surface antigen p30 precursor	22496494
Q27298	P13664	167882	DKKSTAAVILTPTENHFT	129348	Major surface antigen p30 precursor	22496494
Q27298	P13664	167902	FAGAAGSAKSAAGTASHVS	129348	Major surface antigen p30 precursor	22496494
Q27298	P13664	167979	NHFTLKCPKTALTEPPTLA	129348	Major surface antigen p30 precursor	22496494
Q27298	P13664	167988	PIEKFPVTTQTFVVGCIKG	129348	Major surface antigen p30 precursor	22496494
Q27298	P13664	167992	PPTLAYSPNRQICPAGTTS	129348	Major surface antigen p30 precursor	22496494

Table S3a. Experimentally validated peptide-MHC binding related benchmark proteins in Table S1

UniProt ID	Epitope Name	Sub-sequence	Start	End	NCBI GI	Source Molecule	MHC Binding ID	MHC Allele	Qualitative Measurement	Method	PubMed ID
Q27298	T4	VTGLIGSI	324	331	37778533	SAG1 protein	9521	H-2-Kk	Positive	Purified MHC - Radioactivity	10569750
Q27298	SAG1 13-21	GFLTSMFPK	13	21	37778533	SAG1 protein	1245779	HLA-A2	Positive	Lysate - Fluorescence	9240420
Q27298	SAG1 181-189	SSVVNNVAR	181	189	37778533	SAG1 protein	1245780	HLA-A2	Positive	Lysate - Fluorescence	9240420
Q27298	SAG1 213-221	LVCGKDGVK	213	221	37778533	SAG1 protein	1245781	HLA-A2	Positive	Lysate - Fluorescence	9240420
Q27298	SAG1 289-297	SPEKHHCTV	289	297	37778533	SAG1 protein	1245782	HLA-A2	Positive	Lysate - Fluorescence	9240420
Q27298	SAG1 212-220	TLVCGKDGV	212	220	37778533	SAG1 protein	1245806	HLA-A2	Positive	Lysate - Fluorescence	9240420
Q27298	KS9	KSFKDILPK	241	249	37778533	SAG1 protein	1809655	HLA-A*11:01	Positive-Intermediate	Purified MHC - Radioactivity	21129215

Table S3b. Experimentally validated peptide-MHC binding related to homologues of benchmark proteins in Table S1

UniProt ID	Homologue UniProt ID	Epitope Name	Sub-sequence	Start	End	NCBI GI	Source Molecule	MHC Binding ID	MHC Allele	IC_{50}nM	M[2]	PubMed ID
Q27298	P13664	T1	AESKSVII	276	283	129348	Major surface antigen p30 precursor	9515	H-2-Kk	225	R	10569750
Q27298	P13664	T2	QTFVVGCI	155	162	129348	Major surface antigen p30 precursor	9517	H-2-Kk	160	R	10569750
Q27298	P13664	T3	NEKSFKDI	239	246	129348	Major surface antigen p30 precursor	9519	H-2-Kk	123	R	10569750
B6KN48	B5B4W9	SPA 82-90	GLAAAVVAV	78	86	195984531	dense granule antigen precursor	1804405	HLA-A*02:01	9.6	R	21095258
B6KN48	B5B4W9	SPA 82-90	GLAAAVVAV	78	86	195984531	dense granule antigen precursor	1804406	HLA-A*02:02	31	R	21095258

[2] Experimental Method (M): R = Purified MHC - Radioactivity

UniProt ID	Homologue UniProt ID	Epitope Name	Sub-sequence	Start	End	NCBI GI	Source Molecule	MHC Binding ID	MHC Allele	IC_{50}nM	M[1]	PubMed ID
B6KN48	B5B4W9	SPA 82-90	GLAAAVVAV	78	86	195984531	dense granule antigen precursor	1804407	HLA-A*02:03	1.3	R	21095258
B6KN48	B5B4W9	SPA 82-90	GLAAAVVAV	78	86	195984531	dense granule antigen precursor	1804408	HLA-A*02:06	55	R	21095258
B6KN48	B5B4W9	SPA 82-90	GLAAAVVAV	78	86	195984531	dense granule antigen precursor	1804409	HLA-A*68:02	263	R	21095258
B6KN48	B5B4W9	SPA₈₉₋₉₈	AVVSLLRLLK	85	94	195984531	dense granule antigen precursor	1809660	HLA-A*11:01	34	R	21129215
B6KN48	B5B4W9	SPA₈₉₋₉₈	AVVSLLRLLK	85	94	195984531	dense granule antigen precursor	1809782	HLA-A*03:01	17	R	21129215
B6KN48	B5B4W9	SPA₈₉₋₉₈	AVVSLLRLLK	85	94	195984531	dense granule antigen precursor	1809785	HLA-A*30:01	8.1	R	21129215

[1] Experimental Method (M): R = Purified MHC - Radioactivity

UniProt ID	Homologue UniProt ID	Epitope Name	Sub-sequence	Start	End	NCBI GI	Source Molecule	MHC Binding ID	MHC Allele	IC_{50}nM	M[1]	PubMed ID
B6KN48	B5B4W9	SPA₈₉₋₉₈	AVVSLLRLLK	85	94	195984531	dense granule antigen precursor	1809794	HLA-A*68:01	94	R	21129215
A0SJB0	A8I7P3	RS9	RSFKDLLKK	134	142	157824702	granule antigen protein GRA7	1809657	HLA-A*11:01	14	R	21129215
A0SJB0	A0SIX9	A3 GRA7 peptide 3	RSFKDLLKK	134	142	237836631	dense granule protein 7	1847631	HLA-A*03:01	14	R	20347630
A0SJB0	A8I7P3	GRA7 (20-28)	LPQFATAAT	20	28	157824702	granule antigen protein GRA7	1910876	HLA-B*07:02	17	R	22027386
A0SJB0	A0SIX9	A3 GRA7 peptide 3	RSFKDLLKK	134	142	237836631	dense granule protein 7	1847632	HLA-A*11:01	14	R	20347630
A0SJB0	A0SIX9	A3 GRA7 peptide 3	RSFKDLLKK	134	142	237836631	dense granule protein 7	1847633	HLA-A*31:01	303	R	20347630
A0SJB0	A0SIX9	A3 GRA7 peptide 3	RSFKDLLKK	134	142	237836631	dense granule protein 7	1847636	HLA-A*30:01	145	R	20347630

[1] Experimental Method (M): R = Purified MHC - Radioactivity

UniProt ID	Homologue UniProt ID	Epitope Name	Sub-sequence	Start	End	NCBI GI	Source Molecule	MHC Binding ID	MHC Allele	IC_{50}nM	M[1]	PubMed ID
A0SJB0	A0SIX9	B7 GRA7 peptide 2	LPQFATAAT	20	28	237836631	dense granule protein 7	1847643	HLA-B*07:02	14	R	20347630
A0SJB0	A0SIX9	B7 GRA7 peptide 2	LPQFATAAT	20	28	237836631	dense granule protein 7	1847644	HLA-B*35:01	4045	R	20347630
A0SJB0	A0SIX9	B7 GRA7 peptide 2	LPQFATAAT	20	28	237836631	dense granule protein 7	1847646	HLA-B*42:01	17	R	20347630
A0SJB0	A0SIX9	B7 GRA7 peptide 2	LPQFATAAT	20	28	237836631	dense granule protein 7	1847649	HLA-B*54:01	106	R	20347630
B6KEU8	B6KEU8	A2 GRA3 peptide 1	FLVPFVVFL	25	33	308154338	Dense granule protein 3	1847619	HLA-A*02:01	0.1	R	20347630
B6KEU8	B6KEU8	A2 GRA3 peptide 1	FLVPFVVFL	25	33	308154338	Dense granule protein 3	1847620	HLA-A*02:02	0.1	R	20347630
B6KEU8	B6KEU8	A2 GRA3 peptide 1	FLVPFVVFL	25	33	308154338	Dense granule protein 3	1847621	HLA-A*02:03	0.11	R	20347630
B6KEU8	B6KEU8	A2 GRA3 peptide 1	FLVPFVVFL	25	33	308154338	Dense granule protein 3	1847622	HLA-A*02:06	3.5	R	20347630

[1] Experimental Method (M): R = Purified MHC - Radioactivity

UniProt ID	Homologue UniProt ID	Epitope Name	Sub-sequence	Start	End	NCBI GI	Source Molecule	MHC Binding ID	MHC Allele	IC_{50}nM	M[1]	PubMed ID
B6KEU8	B6KEU8	A2 GRA3 peptide 1	FLVPFVVFL	25	33	308154338	Dense granule protein 3	1847623	HLA-A*68:02	1.5	R	20347630
B6KEU8	B6KEU8	B7 GRA3 peptide 3	VPFVVFLVA	27	35	308154338	Dense granule protein 3	1847650	HLA-B*07:02	18	R	20347630
B6KEU8	B6KEU8	B7 GRA3 peptide 3	VPFVVFLVA	27	35	308154338	Dense granule protein 3	1847653	HLA-B*42:01	36	R	20347630
B6KEU8	B6KEU8	B7 GRA3 peptide 3	VPFVVFLVA	27	35	308154338	Dense granule protein 3	1847654	HLA-B*51:01	3016	R	20347630
B6KEU8	B6KEU8	B7 GRA3 peptide 3	VPFVVFLVA	27	35	308154338	Dense granule protein 3	1847656	HLA-B*54:01	0.87	R	20347630
B6KEU8	I7BEN0	GRA7 (27-35)	VPFVVFLVA	27	35	22652337	dense granule protein GRA3	1910877	HLA-B*07:02	18	R	22027386

[1] Experimental Method (M): R = Purified MHC - Radioactivity

6 Printout of evidence profiles used in benchmark dataset

List 1: List of evidence profiles for the test set proteins from Table S1.

Columns: 1= UniProt ID, 2=Phobius_TM, 3=Phobius_SP, 4=SignalP, 5=TargetP_SP, 6=TargetP_loc, 7=TargetP_RC, 8=TMHMM_AA, 9=TMHMM_First60, 10=TMHMM_T M, 11=WoLF_PSORT, 12=WoLF_PSORT_annotation, 13=MHCI, 14= MHCII, 15=Expected classification, 16= Comments (not used by machine learning algorithms)

Surface antigen 1 (SAG1) protein domain is a group of GPI-linked proteins named SRSs (SAG1 related sequence)
Q9UB12,0,Y,0.495,0.708,S,4,13.40,9.13,0,23.0,Secreted,0.5350222,0.3356667,YES,SAG1 Neospora caninum
Q27298,0,Y,0.297,0.141,M,2,7.30,0.56,0,21.5,Secreted,0.2551111,0.2051000,YES,SAG1 (p30)
O96451,1,N,0.218,0.051,M,2,20.48,20.41,1,19.0,Secreted,0.5397556,0.2659667,YES,Surface antigen p35 Neospora caninum
Q9GSE9,2,Y,0.803,0.764,S,3,53.99,16.92,0,11.0,Secreted,0.7648889,0.5968667,YES,Surface antigen p35
Q58L79,1,N,0.218,0.051,M,2,20.48,20.41,1,21.0,Secreted,0.5410889,0.2394000,YES,SRS2 Neospora caninum
B9PVA3,1,Y,0.709,0.977,S,1,33.92,14.86,2,18.5,Secreted,0.3871556,0.4197667,YES,SRS domain containing protein

Dense Granules
B6KEU8,3,N,0.308,0.881,S,2,44.72,22.30,2,9.0,Membrane,0.9105556,0.7197000,YES,GRA3
Q6YDA6,2,Y,0.667,0.886,S,3,33.18,0.67,1,17.0,Secreted,0.6116889,0.3509333,YES,GRA3 Neospora caninum
Q27002,0,Y,0.808,0.952,S,1,10.91,4.48,0,27.0,Secreted,0.8065778,0.7532333,YES,GRA4
B6KN48,2,N,0.779,0.879,S,1,41.56,20.82,2,20.0,Secreted,0.8079111,0.6410000,YES,GRA5
A0SJB0,1,Y,0.906,0.960,S,1,43.90,22.11,2,17.0,Secreted,0.7579778,0.5138000,YES,GRA7
B6KNV3,1,Y,0.803,0.769,S,3,33.08,16.64,0,14.0,Secreted,0.7360000,0.4050333,YES,GRA8
P90661,1,Y,0.793,0.902,S,1,41.04,19.16,2,14.0,Secreted,0.5930667,0.2112333,YES,DG1 Neospora caninum
Q25540,1,Y,0.652,0.740,S,4,39.69,21.75,2,21.0,Secreted,0.6511333,0.5159000,YES,DG2 Neospora caninum
Q9GU48,0,Y,0.841,0.878,S,2,8.95,8.95,0,26.0,Secreted,0.4521111,0.4919000,YES,GRA2 Neospora caninum

Rhoptries
Q04151,0,Y,0.726,0.917,S,1,4.93,4.93,0,26.0,Secreted,0.1767111,0.4246667,YES,ROP1
Q06AK3,0,Y,0.712,0.870,S,2,19.44,18.95,1,14.0,Secreted,0.6178000,0.5832000,YES,ROP2
B6KSS4,0,Y,0.711,0.874,S,2,21.69,18.96,1,14.0,Secreted,0.4215556,0.5068667,YES,ROP4
A7UDC8,0,Y,0.785,0.915,S,1,24.11,18.85,1,19.0,Secreted,0.9402222,0.7485333,YES,ROP
B6KA38,0,N,0.341,0.976,S,1,3.48,3.48,0,17.0,Secreted,0.1964000,0.4824667,YES,ROP8
B6KIB2,1,N,0.263,0.739,S,4,19.46,19.44,1,8.5,Membrane,0.7473556,0.4067667,YES,ROP18
B6KNC1,3,Y,0.763,0.959,S,1,3.50,2.46,0,17.0,Secreted,0.7880000,0.3815667,YES,ROP

Rhoptry neck proteins
Q45WA9,1,N,0.201,0.270,M,4,36.05,20.94,1,17.0,Membrane,0.7089778,0.2132000,YES,RON1
B6KV60,3,Y,0.756,0.946,S,2,71.45,20.65,3,16.0,Membrane,0.8553333,0.6254000,YES,RON2
Q45WA7,3,Y,0.774,0.916,S,2,53.79,10.44,1,30.0,Membrane,0.8822889,0.4592667,YES,RON3
B7UDF2,1,N,0.773,0.892,S,2,17.75,17.74,1,9.0,Secreted,0.8651556,0.4086000,YES,RON8

Micronemes
Q9U483,0,Y,0.427,0.587,S,4,0.23,0.23,0,30.0,Secreted,0.3552667,0.1736000,YES,MIC3 Neospora caninum
Q9GRG4,0,Y,0.696,0.712,S,3,3.02,3.02,0,31.0,Secreted,0.3283333,0.4819333,YES,MIC3
Q8IT72,0,Y,0.769,0.904,S,1,18.30,18.30,1,23.0,Secreted,0.5700000,0.2733333,YES,MIC11 Neospora caninum
B0LUH4,0,Y,0.888,0.907,S,1,0.11,0.11,0,29.0,Secreted,0.2700222,0.3553667,YES,MIC13

Transmembrane proteins
B6KBC5,2,N,0.144,0.327,U,3,29.14,3.36,1,28.0,Membrane,0.4155556,0.4106333,YES,TM
B6KA68,8,N,0.126,0.353,U,3,179.73,2.31,8,31.0,Membrane,0.8706889,0.8324333,YES,TM
B6KVA6,8,N,0.272,0.910,S,2,130.96,19.35,5,23.0,Membrane,0.6701778,0.6755000,YES,TM
B6KIB1,5,N,0.111,0.028,U,2,109.05,0.00,5,30.0,Membrane,0.7994000,0.6625333,YES,TM
B6KMB4,9,N,0.112,0.564,S,5,216.36,34.58,9,32.0,Membrane,0.7898667,0.8300667,YES,TM
Q8MUM2,11,N,0.102,0.100,U,1,264.37,0.05,12,32.0,Membrane,0.8388000,0.5836667,YES,TM
B6KJD0,11,N,0.103,0.038,U,2,236.96,0.00,7,30.0,Membrane,0.5268444,0.7917333,YES,TM

Gliding-associated proteins (GAP)
B9PRA3,10,N,0.120,0.673,S,4,180.32,27.35,9,32.0,Membrane,0.6093778,0.6515667,YES,GAP 45
Q6PQ42,2,Y,0.226,0.195,M,3,44.58,21.39,2,15.0,Membrane,0.5485556,0.3542000,YES,GAP50

Miscellaneous
P84343,0,Y,0.817,0.963,S,1,1.11,1.11,0,29.0,Secreted,0.4652444,0.5364000,YES,Peptidyl-prolyl cis-trans isomerase
B6KA35,11,N,0.101,0.203,U,3,239.31,4.06,11,30.0,Membrane,0.8346667,0.7706333,YES,Nmda receptor glutamate-binding chain
B6KPR6,1,N,0.070,0.249,U,4,20.50,0.00,1,5.0,Membrane,0.5839111,0.4294667,YES,Major sperm protein domain-containing protein
B6KEM3,3,N,0.115,0.510,S,5,62.69,27.24,3,30.0,Membrane,0.5567333,0.6210000,YES,Reticulon domain-containing protein

B6KTX3,2,Y,0.345,0.971,S,1,60.12,29.83,3,15.0,Secreted,0.7359111,0.7550667,YES,TB2/DP1, HVA22 domain-containing protein
B6KJX9,7,Y,0.303,0.303,U,5,138.38,0.10,6,27.0,Membrane,0.8397111,0.7332000,YES,Vacuolar proton-translocating ATPase subunit
B6KBB3,2,N,0.099,0.083,U,2,24.82,0.00,1,4.0,Membrane,0.7684000,0.4860333,YES,Suppressor of actin mutations 2/vacuolar sorting protein
B9PRX5,0,Y,0.250,0.254,M,2,16.81,7.23,0,22.0,Secreted,0.6482889,0.5153667,YES,Proteasome subunit alpha type < FALSE POSITIVE?
B9QH60,1,N,0.322,0.019,M,1,22.02,0.00,1,5.0,Secreted,0.8456889,0.4371667,YES,Acetyl-CoA carboxylase
B6KDM7,0,Y,0.219,0.138,M,2,18.06,17.44,1,13.0,Secreted,0.2830667,0.4517333,YES,Glutamine synthetase< FALSE POSITIVE?
B6KKQ8,1,Y,0.624,0.817,S,3,39.83,18.39,2,9.0,Membrane,0.4822667,0.4643000,YES,Sortilin
B6KAM0,1,Y,0.097,0.372,M,4,31.14,8.76,1,20.5,Membrane,0.3350000,0.3111000,YES,Apical membrane antigen 1
B6KDT4,1,N,0.116,0.026,M,5,38.65,0.51,1,0,NOT_screted_or_membrane,0.6135778,0.3307000,YES,Mitochondrial 2-oxoglutarate/malate carrier
B9PRN1,2,N,0.124,0.248,U,2,60.66,0.72,1,4.0,Membrane,0.6748000,0.8131000,YES,Phosphate carrier protein
B9PRA8,3,N,0.102,0.032,U,1,79.39,0.58,4,0,NOT_screted_or_membrane,0.6952444,0.7165000,YES,ADP/ATP carrier
B9PPS0,3,N,0.333,0.860,S,2,83.69,23.01,4,27.0,Membrane,0.7831111,0.5177333,YES,Thioredoxin

Hypothetical proteins
B9PHB6,3,N,0.302,0.025,M,1,64.94,0.00,3,16.0,Membrane,0.7026667,0.7276000,YES
B9PT54,0,Y,0.892,0.944,S,1,40.79,20.33,2,13.0,Membrane,0.4784000,0.3485667,YES
B9PSP5,0,Y,0.796,0.892,S,2,6.87,3.70,0,14.0,Secreted,0.5633333,0.4086667,YES
B9PKK5,1,N,0.135,0.068,U,2,20.42,0.00,1,3.0,Membrane,0.4862889,0.5146333,YES
B6KH21,1,N,0.255,0.022,M,3,21.20,0.08,1,4.5,Secreted,0.4217778,0.6868333,YES
B6KW78,1,Y,0.742,0.806,S,3,29.91,10.20,1,9.5,Membrane,0.4919111,0.5456333,YES
B6KIG0,1,N,0.181,0.077,U,4,22.91,2.03,1,29.0,Membrane,0.5409778,0.5326333,YES
B6KBB7,2,N,0.175,0.457,U,4,33.65,0.54,0,31.0,Membrane,0.5769111,0.6083667,YES
B6KQ22,1,Y,0.742,0.784,S,3,31.43,11.60,1,0,NOT_screted_or_membrane,0.5864222,0.3353667,YES
B6KVF8,1,N,0.386,0.280,M,4,19.51,19.51,1,5.0,Secreted,0.5101556,0.5859667,YES
B6KC27,8,N,0.141,0.040,M,4,155.01,1.86,7,26.0,Membrane,0.6719778,0.8606667,YES
B6KGA5,8,N,0.108,0.051,U,1,151.24,0.00,7,31.0,Membrane,0.8608889,0.7178000,YES
B6KEH5,9,N,0.108,0.118,U,2,202.95,14.91,9,32.0,Membrane,0.9018667,0.6723667,YES
B6KM08,14,N,0.068,0.055,U,2,315.80,0.00,12,28.0,Membrane,0.8912222,0.5495667,YES
B9PXW3,5,N,0.253,0.932,S,1,94.49,22.25,4,6.5,Secreted,0.5882889,0.5082333,YES

Classification by expert opinion = NO (These proteins are from Che et al., 2010 or Rocchi et al., 2011 studies)
B6KPL7,1,N,0.132,0.018,M,3,9.16,0.01,0,0,NOT_screted_or_membrane,0.7911556,0.6323667,NO
B6KAS6,0,N,0.058,0.075,U,4,0.00,0.00,0,5.0,Secreted,0.4698000,0.3337000,NO
B6KGR8,0,N,0.137,0.088,U,2,0.01,0.00,0,0,NOT_screted_or_membrane,0.3891333,0.3668333,NO
B6KC72,1,N,0.164,0.156,U,5,6.81,0.46,0,5.5,Membrane,0.3953333,0.3842333,NO
B6KAE4,0,N,0.114,0.115,U,2,0.14,0.01,0,3.0,Secreted,0.3036889,0.3765000,NO
B6KHK8,1,N,0.179,0.158,M,2,11.40,11.34,0,6.5,Membrane,0.7855111,0.5479000,NO
B6K9N1,1,N,0.131,0.041,U,2,15.35,0.03,0,5.0,Membrane,0.1972889,0.4806667,NO
B6KC31,0,N,0.137,0.040,M,5,0.00,0.00,0,0,NOT_screted_or_membrane,0.8716000,0.3107667,NO
B6KKV6,0,N,0.153,0.233,U,4,0.11,0.00,0,3.0,Secreted,0.5412889,0.7747667,NO
B6KHJ3,0,N,0.108,0.021,U,4,1.97,0.00,0,5.0,Membrane,0.8399556,0.5709000,NO
B6KPE4,1,N,0.101,0.050,U,1,14.26,0.00,0,0,NOT_screted_or_membrane,0.5333111,0.2045000,NO
B6KPR4,0,N,0.105,0.065,U,1,0.00,0.00,0,0,NOT_screted_or_membrane,0.2112000,0.4071000,NO

Classification by expert opinion = NO (Expected)
B9PGU1,0,N,0.102,0.044,U,1,0.01,0.00,0,0,NOT_screted_or_membrane,0.2223556,0.4924333,NO
B9PVB9,0,N,0.122,0.108,U,2,0.30,0.29,0,5.0,Secreted,0.2191778,0.1158667,NO
B9Q0C2,0,Y,0.245,0.108,U,4,0.54,0.00,0,20.0,Secreted,0.3825111,0.2098333,NO
O02607,0,N,0.127,0.056,U,4,0.04,0.00,0,0,NOT_screted_or_membrane,0.4372000,0.4671667,NO
P52913,0,N,0.108,0.038,U,4,0.61,0.00,0,0,NOT_screted_or_membrane,0.3172667,0.6643333,NO
Q07422,0,N,0.156,0.109,U,4,3.36,0.01,0,0,NOT_screted_or_membrane,0.6512444,0.5775000,NO
Q26998,0,N,0.100,0.076,U,2,0.07,0.00,0,0,NOT_screted_or_membrane,0.4067333,0.2729000,NO
Q27797,0,N,0.171,0.287,U,5,13.35,12.83,0,0,NOT_screted_or_membrane,0.2626444,0.4125667,NO
Q4VRV8,0,N,0.125,0.226,U,2,0.10,0.03,0,0,NOT_screted_or_membrane,0.4350667,0.3453333,NO
Q6QNM1,0,N,0.099,0.107,U,2,1.70,0.57,0,0,NOT_screted_or_membrane,0.3622222,0.4054000,NO
Q8MPF7,0,N,0.107,0.061,U,1,0.05,0.00,0,0,NOT_screted_or_membrane,0.2305333,0.1619000,NO
Q969A8,0,N,0.113,0.073,U,2,0.06,0.00,0,3.0,Secreted,0.1373556,0.1605333,NO
Q9BPL7,0,N,0.105,0.063,U,2,0.72,0.00,0,0,NOT_screted_or_membrane,0.4286889,0.2988000,NO
Q9TVW2,0,N,0.135,0.169,U,3,0.74,0.14,0,13.0,Secreted,0.4202667,0.6241000,NO
Q9UAE6,0,N,0.104,0.079,U,2,0.36,0.00,0,0,NOT_screted_or_membrane,0.3536000,0.4695333,NO
B6KAS0,0,N,0.124,0.158,U,2,0.04,0.00,0,0,NOT_screted_or_membrane,0.3862222,0.3085667,NO
B6KBV1,0,N,0.107,0.052,U,2,0.03,0.00,0,0,NOT_screted_or_membrane,0.3387333,0.4899000,NO
B6KDL6,0,N,0.111,0.064,M,4,0.14,0.00,0,0,NOT_screted_or_membrane,0.3576222,0.6672333,NO
B6KLA4,0,N,0.105,0.055,U,1,0.63,0.01,0,0,NOT_screted_or_membrane,0.2195778,0.3288667,NO
B6KS32,0,N,0.136,0.141,U,2,0.00,0.00,0,0,NOT_screted_or_membrane,0.2620000,0.2931000,NO

B6KT24,0,N,0.102,0.075,U,2,0.34,0.00,0,3.0,Membrane,0.1743778,0.4132000,NO
B9PFW6,0,N,0.111,0.064,M,4,0.14,0.00,0,0,NOT_screted_or_membrane,0.3576222,0.6672333,NO
B9PGU1,0,N,0.102,0.044,U,1,0.01,0.00,0,0,NOT_screted_or_membrane,0.2223556,0.4924333,NO
B9PK71,0,N,0.188,0.223,U,4,0.00,0.00,0,22.0,Secreted,0.3682222,0.3800333,NO
B9PL62,0,N,0.113,0.128,U,2,0.01,0.00,0,3.0,Secreted,0.2566000,0.2519667,NO
B9PLJ9,0,N,0.124,0.158,U,2,0.04,0.00,0,0,NOT_screted_or_membrane,0.3825556,0.3105667,NO
B9PLU2,0,N,0.107,0.064,U,1,0.02,0.00,0,0,NOT_screted_or_membrane,0.3987778,0.1254667,NO
B9PNI2,0,N,0.128,0.037,M,5,0.75,0.41,0,0,NOT_screted_or_membrane,0.4174000,0.2346000,NO
B9PPR0,0,N,0.121,0.091,U,2,0.00,0.00,0,3.0,Secreted,0.2671333,0.1598000,NO
B9PQI4,0,N,0.136,0.141,U,2,0.00,0.00,0,0,NOT_screted_or_membrane,0.2580000,0.2931000,NO
B9PQJ5,0,N,0.119,0.110,U,2,0.23,0.00,0,0,NOT_screted_or_membrane,0.3648667,0.2015000,NO
B9PR77,0,N,0.106,0.055,U,2,0.06,0.05,0,2.0,Secreted,0.2578444,0.2040667,NO
B9PRM0,0,N,0.100,0.039,U,1,0.00,0.00,0,0,NOT_screted_or_membrane,0.1866667,0.5095333,NO
B9PV46,0,N,0.216,0.418,U,3,0.02,0.00,0,0,NOT_screted_or_membrane,0.3103556,0.2669333,NO
B9Q1G8,0,Y,0.167,0.010,M,1,0.00,0.00,0,0,NOT_screted_or_membrane,0.3566444,0.1834667,NO
B9Q1T5,0,N,0.105,0.055,U,1,0.63,0.01,0,0,NOT_screted_or_membrane,0.2025333,0.3288667,NO
B9QAY2,0,N,0.107,0.052,U,2,0.03,0.00,0,0,NOT_screted_or_membrane,0.3387333,0.4899000,NO
B9QC08,0,N,0.124,0.158,U,2,0.04,0.00,0,0,NOT_screted_or_membrane,0.3825556,0.3085667,NO
B9QES2,0,Y,0.165,0.012,M,1,0.00,0.00,0,0,NOT_screted_or_membrane,0.3519333,0.1765333,NO
B9QPA4,0,N,0.105,0.055,U,1,0.63,0.01,0,0,NOT_screted_or_membrane,0.2474222,0.3288667,NO
Q1JTC8,0,N,0.241,0.076,U,4,0.00,0.00,0,0,NOT_screted_or_membrane,0.3693778,0.1680000,NO
Q2Y2R0,0,N,0.106,0.046,U,4,0.15,0.00,0,0,NOT_screted_or_membrane,0.5355111,0.6271333,NO
Q38LF0,0,N,0.115,0.063,U,1,0.00,0.00,0,0,NOT_screted_or_membrane,0.3542444,0.1730000,NO
Q45R46,0,N,0.100,0.034,U,2,0.00,0.00,0,0,NOT_screted_or_membrane,0.1613111,0.4887667,NO
Q4VRV8,0,N,0.125,0.226,U,2,0.10,0.03,0,0,NOT_screted_or_membrane,0.4350667,0.3453333,NO
Q6GW05,0,N,0.101,0.042,U,4,10.78,5.21,0,0,NOT_screted_or_membrane,0.1010444,0.1506000,NO
Q6PWM8,0,N,0.104,0.061,U,3,12.88,5.16,0,0,NOT_screted_or_membrane,0.1118444,0.2288000,NO
Q86RM0,0,N,0.140,0.020,M,3,0.50,0.00,0,0,NOT_screted_or_membrane,0.5237111,0.3294000,NO
Q8MPF7,0,N,0.107,0.061,U,1,0.05,0.00,0,0,NOT_screted_or_membrane,0.2305333,0.1619000,NO
Q8MZS6,0,N,0.098,0.112,U,1,0.28,0.00,0,0,NOT_screted_or_membrane,0.1521778,0.1659333,NO
Q95VT2,0,N,0.108,0.016,U,3,0.00,0.00,0,0,NOT_screted_or_membrane,0.1929333,0.1813667,NO
Q9BPL7,0,N,0.105,0.063,U,2,0.72,0.00,0,0,NOT_screted_or_membrane,0.4286889,0.2988000,NO
Q9NGR6,0,N,0.113,0.314,U,3,0.05,0.00,0,0,NOT_screted_or_membrane,0.2724889,0.2019667,NO
Q9TAP1,0,N,0.140,0.015,M,3,0.20,0.00,0,0,NOT_screted_or_membrane,0.3844000,0.1941667,NO
Q9UAE6,0,N,0.104,0.079,U,2,0.36,0.00,0,0,NOT_screted_or_membrane,0.3536000,0.4695333,NO
Q9XY88,0,N,0.095,0.066,U,1,4.33,0.00,0,3.0,Secreted,0.5446444,0.3026667,NO
Q9NGR7,0,N,0.120,0.096,U,2,0.00,0.00,0,4.0,Secreted,0.3444222,0.1651333,NO
B9Q702,0,N,0.194,0.198,U,3,2.19,0.58,0,6.0,Secreted,0.5166444,0.1010667,NO

7 REFERENCES

1. Horton P, Park K-J, Obayashi T, Fujita N, Harada H, et al. (2007) WoLF PSORT: protein localization predictor. Nucleic Acids Research 35: W585-W587.
2. Petersen TN, Brunak S, von Heijne G, Nielsen H (2011) SignalP 4.0: discriminating signal peptides from transmembrane regions. Nature Methods 8: 785-786.
3. Emanuelsson O, Brunak S, von Heijne G, Nielsen H (2007) Locating proteins in the cell using TargetP, SignalP and related tools. Nat Protocols 2: 953-971.
4. Krogh A, Larsson B, von Heijne G, Sonnhammer ELL (2001) Predicting transmembrane protein topology with a hidden markov model: application to complete genomes. Journal of Molecular Biology 305: 567-580.
5. Kall L, Krogh A, Sonnhammer ELL (2004) A combined transmembrane topology and signal peptide prediction method. Journal of Molecular Biology 338: 1027-1036.
6. Kim Y, Ponomarenko J, Zhu Z, Tamang D, Wang P, et al. (2012) Immune epitope database analysis resource. Nucleic Acids Research 40: W525-W530.
7. Kim Y, Sette A, Peters B (2011) Applications for T-cell epitope queries and tools in the Immune Epitope Database and Analysis Resource. Journal of Immunological Methods 374: 62-69.
8. Dyrløv Bendtsen J, Nielsen H, von Heijne G, Brunak S (2004) Improved Prediction of Signal Peptides: SignalP 3.0. Journal of Molecular Biology 340: 783-795.
9. Emanuelsson O, Nielsen H, Brunak S, von Heijne G (2000) Predicting Subcellular Localization of Proteins Based on their N-terminal Amino Acid Sequence. Journal of Molecular Biology 300: 1005-1016.
10. Nielsen M, Lundegaard C, Worning P, Lauemøller SL, Lamberth K, et al. (2003) Reliable prediction of T-cell epitopes using neural networks with novel sequence representations. Protein Science 12: 1007-1017.
11. Bui H-H, Sidney J, Peters B, Sathiamurthy M, Sinichi A, et al. (2005) Automated generation and evaluation of specific MHC binding predictive tools: ARB matrix applications. Immunogenetics 57: 304-314.
12. Peters B, Sette A (2005) Generating quantitative models describing the sequence specificity of biological processes with the stabilized matrix method. BMC Bioinformatics 6: 132.
13. Sidney J, Assarsson E, Moore C, Ngo S, Pinilla C, et al. (2008) Quantitative peptide binding motifs for 19 human and mouse MHC class I molecules derived using positional scanning combinatorial peptide libraries. Immunome Research 4: 2.
14. Moutaftsi M, Peters B, Pasquetto V, Tscharke DC, Sidney J, et al. (2006) A consensus epitope prediction approach identifies the breadth of murine TCD8+-cell responses to vaccinia virus. Nature biotechnology 24: 817-819.
15. Hoof I, Peters B, Sidney J, Pedersen LE, Sette A, et al. (2009) NetMHCpan, a method for MHC class I binding prediction beyond humans. Immunogenetics 61: 1-13.
16. Wang P, Sidney J, Dow C, Mothe B, Sette A, et al. (2008) A systematic assessment of MHC class II peptide binding predictions and evaluation of a consensus approach. PLoS Computational Biology 4.
17. Nielsen M, Lund O (2009) NN-align. An artificial neural network-based alignment algorithm for MHC class II peptide binding prediction. BMC Bioinformatics 10.
18. Nielsen M, Lundegaard C, Lund O (2007) Prediction of MHC class II binding affinity using SMM-align, a novel stabilization matrix alignment method. BMC Bioinformatics 8: 238.
19. Sturniolo T, Bono E, Ding JY, Raddrizzani L, Tuereci O, et al. (1999) Generation of tissue-specific and promiscuous HLA ligand databases using DNA microarrays and virtual HLA class II matrices. Nature biotechnology 17: 555-561.
20. Bian HJ, Hammer J (2004) Discovery of promiscuous HLA-II-restricted T cell epitopes with TEPITOPE. Methods 34: 468-475.
21. Nielsen M, Justesen S, Lund O, Lundegaard C, Buus S (2010) NetMHCIIpan-2.0 - Improved pan-specific HLA-DR predictions using a novel concurrent alignment and weight optimization training procedure. Immunome Research 6: 9.
22. Rocchi MS, Bartley PM, Inglis NF, Collantes-Fernandez E, Entrican G, et al. (2011) Selection of Neospora caninum antigens stimulating bovine CD4(+ve) T cell responses through immuno-potency screening and proteomic approaches. Veterinary Research 42.
23. Che F-Y, Madrid-Aliste C, Burd B, Zhang H, Nieves E, et al. (2010) Comprehensive proteomic analysis of membrane proteins in toxoplasma gondii. Molecular & Cellular Proteomics.
24. Andrianarivo AG, Anderson ML, Rowe JD, Gardner IA, Reynolds JP, et al. (2005) Immune responses during pregnancy in heifers naturally infected with Neospora caninum with and without immunization. Parasitology Research 96: 24-31.

25. Reichel MP, Ellis JT (2009) Neospora caninum - How close are we to development of an efficacious vaccine that prevents abortion in cattle? International Journal for Parasitology 39: 1173-1187.
26. Tuo WB, Fetterer R, Jenkins M, Dubey JP (2005) Identification and characterization of Neospora caninum cyclophilin that elicits gamma interferon production. Infection and Immunity 73: 5093-5100.
27. Chen Z, Harb OS, Roos DS (2008) In Silico Identification of Specialized Secretory-Organelle Proteins in Apicomplexan Parasites and In Vivo Validation in Toxoplasma gondii. PLoS ONE 3.
28. Keeley A, Soldati D (2004) The glideosome: a molecular machine powering motility and host-cell invasion by apicomplexa. Trends in Cell Biology 14: 528-532.
29. Di Cristina M, Spaccapelo R, Soldati D, Bistoni F, Crisanti A (2000) Two conserved amino acid motifs mediate protein targeting to the micronemes of the apicomplexan parasite Toxoplasma gondii. Molecular and Cellular Biology 20: 7332-7341.
30. Meissner M, Reiss M, Viebig N, Carruthers VB, Toursel C, et al. (2002) A family of transmembrane microneme proteins of Toxoplasma gondii contain EGF-like domains and function as escorters. Journal of Cell Science 115: 563-574.
31. Wootton L (2012) PROTOZOAN PARASITES Directing traffic in apicomplexan parasites. Nature Reviews Microbiology 10.
32. Flower DR, Macdonald IK, Ramakrishnan K, Davies MN, Doytchinova IA (2010) Computer aided selection of candidate vaccine antigens. Immunome Research 6 Suppl 2: S1.
33. Cannas A, Naguleswaran A, Muller N, Eperon S, Gottstein B, et al. (2003) Vaccination of mice against experimental Neospora caninum infection using NcSAG1- and NcSRS2-based recombinant antigens and DNA vaccines. Parasitology 126: 303-312.
34. Howe DK, Crawford AC, Lindsay D, Sibley LD (1998) The p29 and p35 immunodominant antigens of Neospora caninum tachyzoites are homologous to the family of surface antigens of Toxoplasma gondii. Infection and Immunity 66: 5322-5328.
35. Liu K-Y, Zhang D-B, Wei Q-K, Li J, Li G-P, et al. (2006) Biological role of surface Toxoplasma gondii antigen in development of vaccine. World Journal of Gastroenterology 12: 2363-2368.
36. Nishikawa Y, Inoue N, Xuan XN, Nagasawa H, Igarashi I, et al. (2001) Protective efficacy of vaccination by recombinant vaccinia virus against Neospora caninum infection. Vaccine 19: 1381-1390.
37. Bulow R, Boothroyd JC (1991) Protection of mice from fatal *Toxoplasma-gondii* infection by immunization with p-30 antigen in liposomes. Journal of Immunology 147: 3496-3500.
38. Dziadek B, Gatkowska J, Grzybowski M, Dziadek J, Dzitko K, et al. (2012) Toxoplasma gondii: The vaccine potential of three trivalent antigen-cocktails composed of recombinant ROP2, ROP4, GRA4 and SAG1 proteins against chronic toxoplasmosis in BALB/c mice. Experimental Parasitology 131: 133-138.
39. Fux B, Rodrigues CV, Portela RW, Silva NM, Su CL, et al. (2003) Role of cytokines and major histocompatibility complex restriction in mouse resistance to infection with a natural recombinant strain (type I-III) of Toxoplasma gondii. Infection and Immunity 71: 6392-6401.
40. Kasper LH, Khan IA, Ely KH, Buelow R, Boothroyd JC (1992) Antigen-specific (p-30) mouse CD8+ T-cells are cytotoxic against Toxoplasma-gondii-infected peritoneal-macrophages. Journal of Immunology 148: 1493-1498.
41. Li W-S, Chen Q-X, Ye J-X, Xie Z-X, Chen J, et al. (2011) Comparative evaluation of immunization with recombinant protein and plasmid DNA vaccines of fusion antigen ROP2 and SAG1 from Toxoplasma gondii in mice: cellular and humoral immune responses. Parasitology Research 109: 637-644.
42. Lu B, Wu ST, Shi Y, Zhang RL, Zou LJ, et al. (2006) Toxoplasma gondii: Expression pattern and detection of infection using full-length recombinant P35 antigen. Experimental Parasitology 113: 83-90.
43. Baszler TV, Shkap V, Mwangi W, Davies CJ, Mathison BA, et al. (2008) Bovine immune response to inoculation with Neospora caninum surface antigen SRS2 lipopeptides mimics immune response to infection with live parasites. Clinical and Vaccine Immunology 15: 659-667.
44. Cho J-H, Chung W-S, Song K-J, Na B-K, Kang S-W, et al. (2005) Protective efficacy of vaccination with Neospora caninum multiple recombinant antigens against experimental Neospora caninum infection. The Korean journal of parasitology 43: 19-25.
45. Haldorson GJ, Mathison BA, Wenberg K, Conrad PA, Dubey JP, et al. (2005) Immunization with native surface protein NcSRS2 induces a Th2 immune response and reduces congenital Neospora caninum transmission in mice. International Journal for Parasitology 35: 1407-1415.
46. Nishikawa Y, Kousaka Y, Fukumoto S, Xuan XN, Nagasawa H, et al. (2000) Delivery of Neospora caninum surface protein, NcSRS2 (Nc-p43), to mouse using recombinant vaccinia virus. Parasitology Research 86: 934-939.
47. Staska LM, Davies CJ, Brown WC, McGuire TC, Suarez CE, et al. (2005) Identification of vaccine candidate peptides in the NcSRS2 surface protein of Neospora caninum by using CD4(+) cytotoxic T

lymphocytes and gamma interferon-secreting T lymphocytes of infected Holstein cattle. Infection and Immunity 73: 1321-1329.
48. Henriquez FL, Nickdel MB, McLeod R, Lyons RE, Lyons K, et al. (2005) Toxoplasma gondii dense granule protein 3 (GRA3) is a type I transmembrane protein that possesses a cytoplasmic dilysine (KKXX) endoplasmic reticulum (ER) retrieval motif. Parasitology 131: 169-179.
49. Kim JY, Ahn H-J, Ryu KJ, Nam H-W (2008) Interaction between Parasitophorous Vacuolar Membrane-associated GRA3 and Calcium Modulating Ligand of Host Cell Endoplasmic Reticulum in the Parasitism of Toxoplasma gondii. Korean Journal of Parasitology 46: 209-216.
50. Hiszczynska-Sawicka E, Li H, Xu JB, Oledzka G, Kur J, et al. (2010) Comparison of immune response in sheep immunized with DNA vaccine encoding Toxoplasma gondii GRA7 antigen in different adjuvant formulations. Experimental Parasitology 124: 365-372.
51. Liddell S, Parker C, Vinyard B, Jenkins M, Dubey JP (2003) Immunization of mice with plasmid DNA coding for NcGRA7 or NcsHSP33 confers partial protection against vertical transmission of Neospora caninum. Journal of Parasitology 89: 496-500.
52. Liddell S, Lally NC, Jenkins MC, Dubey JP (1998) Isolation of the cDNA encoding a dense granule associated antigen (NCDG2) of Neospora caninum. Molecular and Biochemical Parasitology 93: 153-158.
53. Ellis JT, Ryce C, Atkinson R, Balu S, Jones P, et al. (2000) Isolation, characterization and expression of a GRA2 homologue from Neospora caninum. Parasitology 120: 383-390.
54. Ramamoorthy S, Sanakkayala N, Vemulapalli R, Duncan RB, Lindsay DS, et al. (2007) Prevention of lethal experimental infection of C57BL/6 mice by vaccination with Brucella abortus strain RB51 expressing Neospora caninum antigens. International Journal for Parasitology 37: 1521-1529.
55. Hiszczynska-Sawicka E, Li H, Xu JB, Holec-Gasior L, Kur J, et al. (2011) Modulation of immune response to Toxoplasma gondii in sheep by immunization with a DNA vaccine encoding ROP1 antigen as a fusion protein with ovine CD154. Veterinary Parasitology 183: 72-78.
56. Ossorio PN, Schwartzman JD, Boothroyd JC (1992) A Toxoplasma-gondii rhoptry protein associated with host-cell penetration has unusual charge asymmetry. Molecular and Biochemical Parasitology 50: 1-16.
57. Debache K, Alaeddine F, Guionaud C, Monney T, Mueller J, et al. (2009) Vaccination with recombinant NcROP2 combined with recombinant NcMIC1 and NcMIC3 reduces cerebral infection and vertical transmission in mice experimentally infected with Neospora caninum tachyzoites. International Journal for Parasitology 39: 1373-1384.
58. Carey KL, Jongco AM, Kam K, Ward GE (2004) The Toxoplasma gondii rhoptry protein ROP4 is secreted into the parasitophorous vacuole and becomes phosphorylated in infected cells. Eukaryotic Cell 3: 1320-1330.
59. Beckers CJM, Wakefield T, Joiner KA (1997) The expression of Toxoplasma proteins in Neospora caninum and the identification of a gene encoding a novel rhoptry protein. Molecular and Biochemical Parasitology 89: 209-223.
60. Cheng L, Chen Y, Chen L, Shen Y, Shen J, et al. (2012) Interactions between the ROP18 kinase and host cell proteins that aid in the parasitism of Toxoplasma gondii. Acta Tropica 122: 255-260.
61. El Hajj H, Lebrun M, Arold ST, Vial H, Labesse G, et al. (2007) ROP18 is a rhoptry kinase controlling the intracellular proliferation of Toxoplasma gondii. Plos Pathogens 3: 200-211.
62. Niedelman W, Gold DA, Rosowski EE, Sprokholt JK, Lim D, et al. (2012) The rhoptry proteins ROP18 and ROP5 mediate Toxoplasma gondii evasion of the murine, but not the human, interferon-gamma response. Plos Pathogens 8: e1002784-e1002784.
63. Besteiro S, Michelin A, Poncet J, Dubremetz J-F, Lebrun M (2009) Export of a Toxoplasma gondii Rhoptry Neck Protein Complex at the Host Cell Membrane to Form the Moving Junction during Invasion. Plos Pathogens 5.
64. Bradley PJ, Ward C, Cheng SJ, Alexander DL, Coller S, et al. (2005) Proteomic analysis of rhoptry organelles reveals many novel constituents for host-parasite interactions in Toxoplasma gondii. Journal of Biological Chemistry 280: 34245-34258.
65. Tonkin ML, Roques M, Lamarque MH, Pugniere M, Douguet D, et al. (2011) Host Cell Invasion by Apicomplexan Parasites: Insights from the Co-Structure of AMA1 with a RON2 Peptide. Science 333: 463-467.
66. Sonda S, Fuchs N, Gottstein B, Hemphill A (2000) Molecular characterization of a novel microneme antigen in Neospora caninum. Molecular and Biochemical Parasitology 108: 39-51.
67. Garcia-Reguet N, Lebrun M, Fourmaux MN, Mercereau-Puijalon O, Mann T, et al. (2000) The microneme protein MIC3 of Toxoplasma gondii is a secretory adhesin that binds to both the surface of the host cells and the surface of the parasite. Cellular Microbiology 2: 353-364.
68. Ismael AB, Sekkai D, Collin C, Bout D, Mevelec MN (2003) The MIC3 gene of Toxoplasma gondii is a novel potent vaccine candidate against toxoplasmosis. Infection and Immunity 71: 6222-6228.

69. Harper JM, Zhou XW, Pszenny V, Kafsack BFC, Carruthers VB (2004) The novel coccidian micronemal protein MIC11 undergoes proteolytic maturation by sequential cleavage to remove an internal propeptide. International Journal for Parasitology 34: 1047-1058.
70. Ren D, Zhou D-H, Xu M-J, Zhou Y, Yang J-F, et al. (2012) Sequence variation in Toxoplasma gondii MIC13 gene among isolates from different hosts and geographical locations. African Journal of Microbiology Research 6: 3265-3269.
71. Ortega-Barria E, Boothroyd JC (1999) A Toxoplasma lectin-like activity specific for sulfated polysaccharides is involved in host cell infection. Journal of Biological Chemistry 274: 1267-1276.
72. Lourenco EV, Pereira SR, Faca VM, Coelho-Castelo AAM, Mineo JR, et al. (2001) Toxoplasma gondii micronemal protein MIC1 is a lactose-binding lectin. Glycobiology 11: 541-547.
73. Massimine KM, Doan LT, Atreya CA, Stedman TT, Anderson KS, et al. (2005) Toxoplasma gondii is capable of exogenous folate transport - A likely expansion of the BT1 family of transmembrane proteins. Molecular and Biochemical Parasitology 144: 44-54.
74. Blume M, Rodriguez-Contreras D, Landfear S, Fleige T, Soldati-Favre D, et al. (2009) Host-derived glucose and its transporter in the obligate intracellular pathogen Toxoplasma gondii are dispensable by glutaminolysis. Proceedings of the National Academy of Sciences of the United States of America 106: 12998-13003.
75. Stedman TT, Sussman R, Joiner KA (2001) Intracellular trafficking of a Toxoplasma gondii glucose transporter is regulated by NSF and Rab11. Molecular Biology of the Cell 12: 518A-518A.
76. Nagamune K, Sibley LD (2006) Comparative genomic and phylogenetic analyses of calcium ATPases and calcium-regulated proteins in the apicomplexa. Molecular Biology and Evolution 23: 1613-1627.
77. Frenal K, Polonais V, Marq J-B, Stratmann R, Limenitakis J, et al. (2010) Functional Dissection of the Apicomplexan Glideosome Molecular Architecture. Cell Host & Microbe 8: 343-357.
78. Gaskins E, Gilk S, DeVore N, Mann T, Ward G, et al. (2004) Identification of the membrane receptor of a class XIV myosin in Toxoplasma gondii. Journal of Cell Biology 165: 383-393.
79. Diakov TT, Kane PM (2010) Regulation of vacuolar proton-translocating ATPase activity and assembly by extracellular pH. The Journal of biological chemistry 285: 23771-23778.
80. Bishop N, Woodmane P (2000) ATPase-defective mammalian VPS4 localizes to aberrant endosomes and impairs cholesterol trafficking. Molecular Biology of the Cell 11: 227-239.
81. Jelenska J, Crawford MJ, Harb OS, Zuther E, Haselkorn R, et al. (2001) Subcellular localization of acetyl-CoA carboxylase in the apicomplexan parasite Toxoplasma gondii. Proceedings of the National Academy of Sciences of the United States of America 98: 2723-2728.
82. Zuther E, Johnson JJ, Haselkorn R, McLeod R, Gornicki P (1999) Growth of Toxoplasma gondii is inhibited by aryloxyphenoxypropionate herbicides targeting acetyl-CoA carboxylase. Proceedings of the National Academy of Sciences of the United States of America 96: 13387-13392.
83. Sloves P-J, Delhaye S, Mouveaux T, Werkmeister E, Slomianny C, et al. (2012) Toxoplasma Sortilin-like Receptor Regulates Protein Transport and Is Essential for Apical Secretory Organelle Biogenesis and Host Infection. Cell Host & Microbe 11: 515-527.
84. Hehl AB, Lekutis C, Grigg ME, Bradley PJ, Dubremetz JF, et al. (2000) Toxoplasma gondii homologue of plasmodium apical membrane antigen 1 is involved in invasion of host cells. Infection and Immunity 68: 7078-7086.
85. Parussini F, Tang Q, Moin SM, Mital J, Urban S, et al. (2012) Intramembrane proteolysis of Toxoplasma apical membrane antigen 1 facilitates host-cell invasion but is dispensable for replication. Proceedings of the National Academy of Sciences of the United States of America 109: 7463-7468.
86. Zhang H, Compaore MKA, Lee E-g, Liao M, Zhang G, et al. (2007) Apical membrane antigen 1 is a cross-reactive antigen between Neospora caninum and Toxoplasma gondii, and the anti-NcAMA1 antibody inhibits host cell invasion by both parasites*. Molecular and Biochemical Parasitology 151: 205-212.
87. DeRocher AE, Coppens I, Karnataki A, Gilbert LA, Rome ME, et al. (2008) A thioredoxin family protein of the apicoplast periphery identifies abundant candidate transport vesicles in Toxoplasma gondii. Eukaryotic Cell 7: 1518-1529.
88. Howe DK, Sibley LD (1999) Comparison of the major antigens of Neospora caninum and Toxoplasma gondii. International Journal for Parasitology 29: 1489-1496.
89. Hemphill A (1996) Subcellular localization and functional characterization of Nc-p43, a major Neospora caninum tachyzoite surface protein. Infection and Immunity 64: 4279-4287.
90. Hemphill A, Felleisen R, Connolly B, Gottstein B, Hentrich B, et al. (1997) Characterization of a cDNA-clone encoding Nc-p43, a major Neospora caninum tachyzoite surface protein. Parasitology 115: 581-590.
91. Haldorson GJ, Stanton JB, Mathison BA, Suarez CE, Baszler TV (2006) Neospora caninum: Antibodies directed against tachyzoite surface protein NcSRS2 inhibit parasite attachment and invasion of placental trophoblasts in vitro. Experimental Parasitology 112: 172-178.

92. Sonda S, Fuchs N, Connolly B, Fernandez P, Gottstein B, et al. (1998) The major 36 kDa Neospora caninum tachyzoite surface protein is closely related to the major Toxoplasma gondii surface antigen. Molecular and Biochemical Parasitology 97: 97-108.
93. Hemphill A, Fuchs N, Sonda S, Gottstein B, Hentrich B (1997) Identification and partial characterization of a 36 kDa surface protein on Neospora caninum tachyzoites. Parasitology 115: 371-380.
94. Jung C, Lee CYF, Grigg ME (2004) The SRS superfamily of Toxoplasma surface proteins. International Journal for Parasitology 34: 285-296.
95. Papadopoulou B, Roy G, Breton M, Kundig C, Dumas C, et al. (2002) Reduced infectivity of a Leishmania donovani biopterin transporter genetic mutant and its use as an attenuated strain for vaccination. Infection and Immunity 70: 62-68.
96. Ramey K, Eko FO, Thompson WE, Armah H, Igietseme JU, et al. (2009) Immunolocalization and Challenge Studies Using a Recombinant Vibrio cholerae Ghost Expressing Trypanosoma brucei Ca2+ ATPase (TBCA2) Antigen. American Journal of Tropical Medicine and Hygiene 81: 407-415.
97. Joet T, Eckstein-Ludwig U, Morin C, Krishna S (2003) Validation of the hexose transporter of Plasmodium falciparum as a novel drug target. Proceedings of the National Academy of Sciences of the United States of America 100: 7476-7479.
98. Ellis J, Miller C, Quinn H, Ryce C, Reichel MP (2008) Evaluation of recombinant proteins of Neospora caninum as vaccine candidates (in a mouse model). Vaccine 26: 5989-5996.
99. Dubremetz JF, Garcia-Reguet N, Conseil V, Fourmaux MN (1998) Apical organelles and host-cell invasion by Apicomplexa. International Journal for Parasitology 28: 1007-1013.
100. Debache K, Guionaud C, Alaeddine F, Mevissen M, Hemphill A (2008) Vaccination of mice with recombinant NcROP2 antigen reduces mortality and cerebral infection in mice infected with Neospora caninum tachyzoites. International Journal for Parasitology 38: 1455-1463.
101. Sher A, Denkers EY, Gazzinelli RT (1995) Induction and regulation of host cell-mediated immunity by Toxoplasma gondii. In: Chadwick D, Cardew G, editors. T Cell Subsets in Infectious and Autoimmune Diseases. pp. 95-104.
102. Vaughan AM, Wang R, Kappe SHI (2010) Genetically engineered, attenuated whole-cell vaccine approaches for malaria. Human Vaccines 6: 107-113.
103. Mazumdar J, Wilson EH, Masek K, Hunter CA, Striepen B (2006) Apicoplast fatty acid synthesis is essential for organelle biogenesis and parasite survival in Toxoplasma gondii. Proceedings of the National Academy of Sciences of the United States of America 103: 13192-13197.
